# Comprehensive lung microbial gene and genome catalogs assist the mechanism survey of *Mesomycoplasma hyopneumoniae* strains causing pig lung lesions

**DOI:** 10.1002/imt2.258

**Published:** 2024-12-26

**Authors:** Jingquan Li, Fei Huang, Yunyan Zhou, Tao Huang, Xinkai Tong, Mingpeng Zhang, Jiaqi Chen, Zhou Zhang, Huipeng Du, Zifeng Liu, Meng Zhou, Yiwen Xiahou, Huashui Ai, Congying Chen, Lusheng Huang

**Affiliations:** ^1^ National Key Laboratory of Pig Genetic Improvement and Germplasm Innovation Jiangxi Agricultural University Nanchang China

**Keywords:** adhesion, lung lesions, lung microbiome, *Mesomycoplasma hyopneumoniae*, metagenome‐assembled genomes, pig

## Abstract

Understanding the community structure of the lower respiratory tract microbiome is crucial for elucidating its roles in respiratory tract diseases. However, there are few studies about this topic due to the difficulty in obtaining microbial samples from both healthy and disease individuals. Here, using 744 high‐depth metagenomic sequencing data of lower respiratory tract microbial samples from 675 well‐phenotyped pigs, we constructed a lung microbial gene catalog containing the largest scale of 10,031,593 nonredundant genes to date, 44.8% of which are novel. We obtained 356 metagenome‐assembled genomes (MAGs) which were further clustered into 256 species‐level genome bins with 41.8% being first reported in the current databases. Based on these data sets and through integrated analysis of the isolation of the related bacterial strains, in vitro infection, and RNA sequencing, we identified and confirmed that *Mesomycoplasma hyopneumoniae* (*M. hyopneumoniae*) MAG_47 and its adhesion‐related virulence factors (VFs) were associated with lung lesions in pigs. Differential expression levels of adhesion‐ and immunomodulation‐related VFs likely determined the heterogenicity of adhesion and pathogenicity among *M. hyopneumoniae* strains. *M. hyopneumoniae* adhesion activated several pathways, including nuclear factor kappa‐light‐chain‐enhancer of activated B, mitogen‐activated protein kinase, cell apoptosis, T helper 1 and T helper 2 cell differentiation, tumor necrosis factor signaling, interleukin‐6/janus kinase 2/signal transducer and activator of transcription signaling, and response to reactive oxygen species, leading to cilium loss, epithelial cell‒cell barrier disruption, and lung tissue lesions. Finally, we observed the similar phylogenetic compositions of the lung microbiome between humans with *Mycoplasma pneumoniae* and pigs infected with *M. hyopneumoniae*. The results provided important insights into pig lower respiratory tract microbiome and its relationship with lung health.

## Highlights

- We constructed a comprehensive microbial gene catalog of lower respiratory tract microbiome, containing 10,031,593 nonredundant genes and obtained 356 nonredundant metagenome‐assembled genomes using five representative pig populations.
- Significant associations of *Mesomycoplasma hyopneumoniae* (*M. hyopneumoniae*) strains and their adhesion‐related virulence factors with lung lesions were observed and confirmed in two different pig populations, as well as through in vitro infection of porcine bronchial epithelial cells.
- *M. hyopneumoniae* adhesion activated several pathways, such as nuclear factor kappa‐light‐chain‐enhancer of activated B, mitogen‐activated protein kinase, cell apoptosis and response to reactive oxygen species, which resulted in cilium loss, epithelial cell‒cell barrier disruption, and lung tissue lesions.

## Graphical abstract

In this study, we utilized 744 metagenomic sequencing data from lower respiratory tract microbial samples of 675 well‐phenotyped pigs across five cohorts. From these, we constructed a microbial gene catalog containing 10,031,593 nonredundant genes and 356 metagenome‐assembled genomes (MAGs), which were further clustered into 256 species‐level genome bins. Based on these data sets, we observed the significant association between *Mesomycoplasma hyopneumoniae* (*M. hyopneumoniae*) and lung lesions at the strain level. This association was confirmed by in vitro infection of porcine bronchial epithelial cells (BECs) with two *M. hyopneumoniae strains*. By employing dual RNA sequencing of both *M. hyopneumoniae* and infected BECs, alongside RNA‐sequencing data from two pure‐cultured *M. hyopneumoniae* strains, we elucidated the mechanism of *M. hyopneumoniae* causing pig lung lesions and explained the observed heterogeneity in pathogenicity among *M. hyopneumoniae* strains.

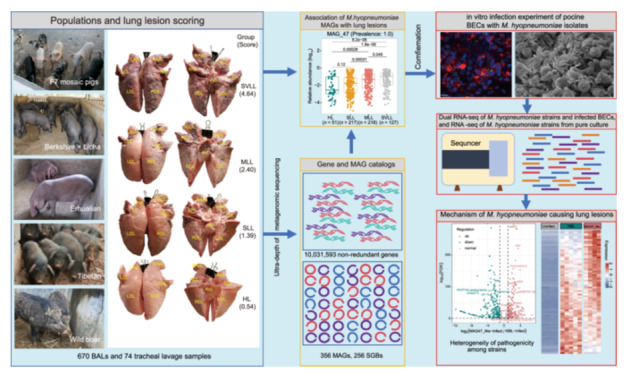

## INTRODUCTION

Respiratory diseases, which influence pig growth and feed conversion efficiency and result in significant economic losses, are considered among the most important health problems in pig production [1]. The diagnosis of respiratory diseases in pigs is often challenging and relies on a combination lung lesion evaluation and abattoir inspection. Respiratory diseases in pigs are multifactorial and are often the result of the interplay of infectious agents and environmental factors, such as ventilation, production systems, and management methods [2, 3]. In the past few years, growing evidence has suggested that the lung microbiome has a significant impact on respiratory diseases [4, 5]. It is thus critical to reveal the community structure of the lung microbiome to explain its roles in maintaining lung health and in related diseases.

Although the diversity and abundance of the lung microbiota are much lower than those of the oral and gut microbiota, culture‐independent techniques, such as 16S rRNA gene sequencing, have demonstrated that the lung microbiota is diverse and highly individual specific [6]. The utilization of shotgun metagenomic sequencing makes it possible to infer the functional capacities of microbial communities and to explore the microbial species associated with lung diseases at the strain level. To date, metagenomic sequencing has been used to establish a respiratory tract microbial gene catalog [7] and to diagnose lung diseases and respiratory infections in humans [8]. However, a comprehensive catalog of reference genes/genomes of the respiratory tract microbiome from a broad range of sample sources is lacking due to the collection of bronchoalveolar lavage (BAL) fluid samples from participants is highly difficult in studies of the human lung microbiota. To our knowledge, systematic studies of microbial composition at the species level and the functional capacities of the lung microbiome are notably lacking in pigs. This has severely limited the use of metagenomic sequencing in the analysis of pig lower respiratory tract microbial communities and in the diagnosis of respiratory diseases. Furthermore, several studies have suggested that *Mesomycoplasma hyopneumoniae* (*M. hyopneumoniae*) is one of the primary agents involved in the porcine chronic respiratory disease complex (enzootic pneumonia) [9, 10, 11, 12]. However, a clear mechanism of *M. hyopneumoniae* pathogenicity has not been identified. Several studies in humans have also shown that *Mycoplasma pneumoniae* is one of the leading bacterial pathogens associated with acute respiratory infections in humans [7, 13]. Considering the high anatomical similarities between human and porcine lungs, whether the microbial composition and potential functional capacities of the lung microbiome are similar in individuals infected with *Mesomycoplasma* or with inflammatory lung diseases between humans and pigs, is largely unknown.

In this study, given the advantage that microbial samples could be obtained immediately from pig lungs after slaughter to effectively avoid contamination from the mouth and upper respiratory tract, we performed metagenomic sequencing on 744 microbial samples collected from the lower respiratory tract of five pig breeds. We constructed a comprehensive pig lower respiratory tract gene catalog (PRGC) containing 10,031,593 genes and obtained 356 metagenome‐assembled genomes (MAGs) by binning analysis. Based on this, we characterized the community structure of the porcine lower respiratory tract microbiome at high resolution, confirmed the role of *M. hyopneumoniae* strains in lung lesions, which are considered a trait reflecting the status of lung diseases, and explored the mechanism by which *M. hyopneumoniae* strains cause lung lesions by using in vitro infection experiments with bronchial epithelial cells (BECs) and gene expression analysis.

## RESULTS

### Construction of a comprehensive gene catalog of the porcine lower respiratory tract microbiome

We collected 744 microbial samples comprising 670 BAL samples and 74 tracheal lavage samples from 675 pigs from five broadly representative pig populations within 30 min after slaughter (see Methods) (Tables S1 and S2). Shotgun metagenomic sequencing generated an average of 70.30 Gbp of raw sequencing data per sample (Figure S1A). After quality control and removal of host DNA contamination, an average of 1.52 Gbp of clean reads per sample was obtained for subsequent analyses (Figure S1B). Six blank samples of phosphate‐buffered saline (PBS) solution that were used for lavage and six sequencing background control samples from mixed reagents were sequenced (see Methods). Under the same sequencing conditions, significantly low amounts of sequencing data were obtained for these control samples (28.4 ~ 473.2 Mb of raw data) (Figure S1C). We identified 10 bacterial species whose total abundance accounted for an average of 97.9% of the total microbial abundance in the control samples. However, only three out of these 10 species were also identified in the 744 tested samples (Table S3). Moreover, their total relative abundances were 0.02% on average (ranging from 0% to 1.2%) in tested samples (Table S4). This suggested that contamination was unlikely to have a substantial influence on the observed microbiome profiles.

**Figure 1:**
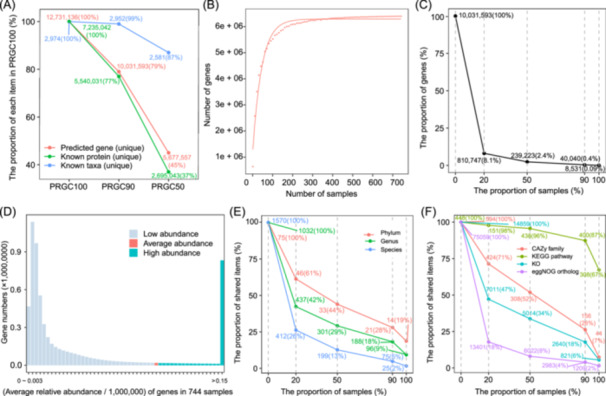
Construction of a comprehensive gene catalog of the porcine lower respiratory tract microbiome and its profiles of taxonomic compositions and potential functional capacities. (A) The numbers (percentages) of predicted nonredundant genes, known proteins, and taxa in three gene catalogs removed redundant genes according to 100.0%, 90.0%, and 50.0% amino acid sequence identity. The percentages in the brackets are the proportions of each item in pig lower respiratory tract gene catalog at 100% amino acid sequence identity (PRGC100). The protein sequences or bacterial taxa that could be aligned to the UniProt TrEMBL database were defined as known proteins and taxa. (B) Accumulation curve showing that the number of nonredundant genes increased with increasing sample size in the PRGC90. Dots near the curve represent the average values of 10 bootstraps. (C) The numbers (percentages) of nonredundant genes in PRGC90 shared across different proportions of 744 samples. (D) The number of genes in each range of relative abundances. The x‐axis shows the average relative abundance of genes in the 744 samples (relative abundance/10^6^). The different colored bars indicate the number of genes in each relative abundance interval (gray bars: under the average abundance; red bars: at the average abundance; blue bars: above the average abundance). (E) The numbers (percentages) of shared taxa among different proportions of samples at the phylum (red), genus (green), and species (blue) levels. (F) The numbers (percentages) of shared functional items in each proportion of samples for the carbohydrate‐active enzyme (CAZy) family (red), Kyoto Encyclopedia of Genes and Genomes (KEGG) pathways (olive), KEGG orthologues (cyan), and evolutionary genealogy of genes: Nonsupervised Orthologous Group (eggNOG) orthologs (purple). The values next to the dots represent the numbers and proportions of items in PRGC90 shared by 20.0%, 50.0%, 90.0%, and 100.0% of the tested samples. The x‐axis indicates the proportions of samples, and the y‐axis shows the proportions of shared items.

We implemented an assembly strategy combining both single‐sample assembly and co‐assembly of all sequenced samples. A total of 19,365,625 contigs with a length ≥500 bp were obtained. On average, over 81.2% of clean reads in each sample could be aligned to these contigs. All predicted genes from assembled contigs were first filtered with the threshold of length >100 bp. Genes with a complete open reading frame (ORF) in the Prodigal prediction were retained even if their lengths were <100 bp. All genes were then clustered to construct a nonredundant gene catalog according to their amino acid sequences at 100.0%, 90.0%, and 50.0% sequence identity, resulting in the gene catalogs PRGC100, PRGC90, and PRGC50, respectively. We further filtered out those genes that were annotated to eukaryotes, while genes classified as fungi and protists were retained, which were important members of the lower respiratory microecosystem. After that, the numbers of genes in the three gene catalogs were as follows: 12,731,136 (PRGC100), 10,031,593 (PRGC90), and 5,677,557 (PRGC50) (Figure 1A). Compared to the PRGC100, the number of annotated proteins in the PRGC90 and PRGC50 was decreased by 21.0% and 55.0%, respectively. However, the number of identified taxa was only lowered 1.0% in the PRGC90 (Figure 1A), whereas it decreased by 13.0% in the PRGC50, indicating that those removed genes at 90.0% of sequence identity threshold were predominantly redundant. Dereplication did not significantly affect the identification of microbial taxa. Thus, the PRGC90 catalog was used for further analyses.

Rarefaction curves based on the PRGC90 showed that the number of genes tended to be saturated when the number of samples was ≥200 (Figure 1B). Compared to the 10,031,593 genes in PRGC90, the number of genes at saturation was more than six million. This finding suggested that the co‐assembly strategy could ensure the acquisition of many genes that could not be assembled in a single sample because of low abundance. This condition can be expected under insufficient sequencing depth [14]. We then investigated the prevalence of predicted genes in PRGC90 across the 744 samples. The number of genes present in at least one sample was 6,398,682 (63.8%). There were 810,747 (8.1%) genes that existed in >20.0% of the samples, and only 40,040 (0.4%) genes were present in >90.0% of all the tested samples (Figure 1C). In addition, we further analyzed the relative abundance of 6.40 million genes that existed in at least one sample and found that most of these genes exhibited low abundance. Only 1,079,287 genes had an abundance greater than the average abundance (Figure 1D). These results suggested high diversity and heterogeneity of microbial gene compositions in the swine lower respiratory tract microbiome.

### Profiling the taxonomic compositions and potential functional capacities of the porcine lower respiratory tract microbiome based on PRGC90

The phylogenetic composition of the porcine lower respiratory tract microbiome was determined by mapping genes in PRGC90 to the UniProt TrEMBL database. A total of 3,179,743 (31.7%) genes could be annotated taxonomically. These genes were assigned to four kingdoms, 81 phyla, 1018 genera, and 1570 unique species. Among the 1570 microbial species, 1429 were bacteria. At the phylum level, Proteobacteria (44.0%), Tenericutes (31.0%), Firmicutes (10.0%), Bacteroidetes (6.0%), and Actinobacteria (4.0%) were predominant in the porcine lower respiratory tract microbiome. A total of 11 phyla, 124 genera, and 63 species were identified as viral taxa. Among these viral taxa, *Ungulate tetraparvovirus 2* had the highest abundance (Figure S2A). There were four phyla, 38 genera, and 21 species of fungi. *Pneumocystis jirovecii* was the dominant fungal species (Figure S2B). We also identified nine species belonging to three phyla and nine genera of archaea. Among them, *Methanobrevibacter smithii* was predominant (Figure S2C). Moreover, we investigated the prevalence of these annotated taxa among the 744 tested samples at the phylum, genus, and species levels. Twenty‐one phyla (28.0% of all phyla identified) were detected in more than 90.0% of the tested samples (Figure 1E). In contrast, only 188 genera (18.0%) and 75 species (5.0%) were detected in >90.0% of the tested samples, further suggesting heterogeneity in the microbial composition of the swine lower respiratory tract microbiome. Notably, most of the microbial taxa (>97.0% species) with a high prevalence were bacteria. Virus, fungal, and archaeal species accounted for only 0.6% of the total abundance (Figure S2D), so we focused on bacterial taxa in the subsequent analyses.

The potential functional capacities of the porcine lower respiratory tract microbiome were determined by annotating genes in PRGC90 to the carbohydrate‐active enzyme (CAZyme), Kyoto Encyclopedia of Genes and Genomes (KEGG), and evolutionary genealogy of genes: Nonsupervised Orthologous Group (eggNOG) databases. A total of 154,154 genes were annotated to 594 unique CAZy families, 3,078,547 genes to 458 KEGG pathways, and 4,362,614 genes to 75,059 eggNOG orthologs (Table S5). Similar to the prevalence of microbial taxa, 26.0% of the CAZy families, 18.0% of the KEGG orthologs (KOs), 67.0% of the KEGG pathways, and 4.0% of the eggNOG orthologs were present in more than 90.0% of all the tested samples (Figure 1F). The low percentages of shared functional capacities indicated the heterogeneity of the functional capacities of the porcine lower respiratory microbiome.

### Constructing MAGs of the swine lower respiratory tract microbiome

Because of the high proportion of host DNA contamination in lower respiratory tract microbial samples [15], few studies have been able to reconstruct microbial genomes from metagenomic sequencing data of the lower respiratory tract microbiome. In this study, 1965 MAGs with completeness ≥50.0% and contamination ≤10.0% were recovered by a metagenomic binning procedure using contigs generated by both single‐sample assembly and co‐assembly. The 1965 MAGs were clustered into 356 nonredundant MAGs based on 99.0% average nucleotide identity (ANI) (Table S6). Among these 356 MAGs, 260 MAGs were of medium quality (at least 50.0% completeness and <10.0% contamination), including 194 MAGs with a quality score (QS) ≥ 50 (completeness—5 × contamination) [16] and one MAG with completeness ≥90.0% but contamination >5.0%. The other 96 MAGs were near‐complete (completeness ≥90.0% and contamination ≤5.0%) (Figure S3), among which 19 MAGs had the 5S, 16S, 23S rRNA genes and at least 18 tRNAs that met the MIMAG standards for “high‐quality” MAGs set by the Genomic Standards Consortium [17]. Prevalence analysis revealed that most of the MAGs had a low prevalence in the 744 metagenomic sequencing samples. Only eight MAGs were identified in >90.0% of the tested samples, and 170 MAGs were present in at least 20.0% of the tested samples, further suggesting the high heterogeneity of the microbial composition of the porcine lower respiratory tract microbiome across samples. Certainly, different sequencing depths across samples and the complexity of the assembly process for MAG reconstruction could not be excluded.

**Figure 2:**
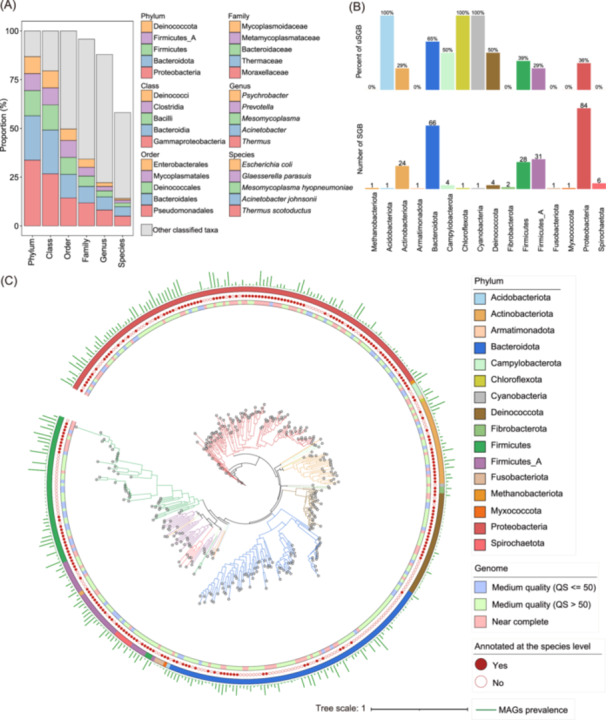
Constructing metagenome‐assembled genomes (MAGs) of the swine lower respiratory tract microbiome. (A) Taxonomic composition of 356 nonredundant MAGs constructed in this study. The colored boxes indicate the proportion of MAGs annotated to each taxon. Only five taxa with the highest proportions of MAGs at each level are shown, and the other taxa were classified as “other classified taxa.” (B) The number of species‐level genome bins (SGBs) in each phylum and the percentage of unknown SGBs (uSGBs) in each phylum. The SGBs that could not be matched to any reference genomes according to the GTDB‐tk were defined as uSGBs. (C) Phylogenetic tree of 356 MAGs in the swine lower respiratory tract microbiome. From the inner to outer circles, the first circle indicates the quality of MAGs (medium quality quality score [QS] ≤ 50), medium quality [QS > 50] or near‐complete), the second circle shows whether the MAGs could be annotated to a species or not, the third circle indicates the corresponding phyla which MAGs belonged to, and the green bars in the fourth layer represent the prevalence of MAGs in 744 tested samples.

All 356 nonredundant MAGs were subjected to taxonomic annotation using GTDB‐Tk [18]. Among them, 355 MAGs were classified as bacteria (15 phyla), and the other MAG was annotated to *Methanobrevibacter A sp900769095* (Methanobacteriota), a methanogenic archaeon. Approximately 58.2% (207) of the MAGs could be assigned to 149 unique species (Figure 2A,B). The largest number of MAGs (120) was assigned to Proteobacteria, followed by Bacteroidetes (81) and Firmicutes (46). To determine the phylogenetic relationships of these MAGs, a maximum likelihood phylogenetic tree of the 356 MAGs was constructed by PhyloPhlAn based on 400 universal marker genes (Figure 2C). The MAGs belonging to Proteobacteria were widely distributed in the phylogenetic tree, indicating high phylogenetic diversity.

All 356 MAGs were further clustered into 256 species‐level genome bins (SGBs) at the 95.0% ANI threshold. Among these 256 SGBs, 41.8% (107) did not match any available genomes within GTDB‐Tk and therefore were defined as unknown SGBs (uSGBs) (Figure 2B). There were 84, 66, and 31 SGBs belonging to Proteobacteria, Bacteroidota, and Firmicutes_A, respectively. The SGBs annotated to *Thermus scotoductus*, *Acinetobacter johnsonii*, and *M. hyopneumoniae* contained the greatest numbers of MAGs (18, 17, and 7, respectively). All 107 uSGBs could be assigned at the order level, and 80.0% were annotated at the genus level, suggesting that a substantial portion of uSGBs might be novel bacterial species.

### Identifying virulence factor genes (VFGs) and their host bacteria by integrating microbial gene and MAG catalogs

By a BLAST search against the Virulence Factor Database (VFDB) [19], we identified 778,009 ORFs encoding virulence factors (VFs) in PRGC90. These ORFs could be classified into 17,144 VFGs belonging to 1162 VF types (e.g., P97 and Hemolysin) and 14 functional VF categories (e.g., adherence and exotoxin). The main functional categories of these 17,144 VFGs included immune modulation (20.3% of the total number), effector delivery system (15.8%), nutritional/metabolic factor (15.5%), adherence (12.8%), and motility (12.3%) (Table S7). We further aligned the sequences of VFGs to the UniProt TrEMBL database to identify their potential host bacteria. At the genus level, 17,144 VFGs were widely distributed in 559 bacterial genera belonging to 30 phyla, especially *Pseudomonas* and *Acinetobacter*. At the species level, a total of 639 host bacterial species were identified (Table S8). The taxonomic distribution of the host species in which the numbers of VFGs they harbored were ranked in the top 50 is shown in Figure S4. *Moraxella osloensis* carried the largest number of VFGs (3014). We performed an in‐depth analysis of the distribution of VFGs among the MAGs. A total of 10,845 VFGs were identified among the 356 MAGs. The greatest number of VFGs (1028) was detected in MAG_155, which was annotated to *Mesorhizobium*.

### Comparison of core microbial species in the lower respiratory microbiome between two respiratory tract locations and among five pig populations

There were 69 pigs from which both tracheal and BAL fluid samples were collected. This facilitated the comparison of the microbial compositions between tracheal and BAL fluid samples. The microbial composition of the tracheal samples showed greater *α* diversity based on the observed species index, although the difference in the Shannon index was not significant (Figure S5A). The *β* diversity of these samples was also significantly greater than that of BAL fluid samples based on the Bray‒Curtis distance and principal coordinate analysis (PCoA) (*p* = 9.3 × 10^−4^) (Figure S5B). At the taxonomic level, seven core species whose relative abundances were listed in the top 20 were specifically identified in each of the tracheal and BAL fluid sample types (Figure S5C).

We then explored the distribution of the core species of the lung microbiome in each of the five pig populations used in this study. The microbial species detected in >95.0% of individuals in each population were defined as core species. A total of 65, 70, 74, 75, and 116 core species were identified in the F_7_, Berkshire × Licha, Tibetan, and Erhualian pigs and wild boars, respectively. We focused on those core species whose abundances were ranked in the top 20 in each population. *M. hyopneumoniae*, *M. osloensis*, *Prevotella copri*, *Escherichia coli*, and *Glaesserella parasuis* were identified in the top 20 lists for all five populations. The Chinese indigenous Erhualian (five species) and Tibetan (seven species) pigs and wild boars (eight species) had more population‐specific core species (Figure S6).

### Identifying bacterial taxa and potential functional capacities associated with lung lesions

**Figure 3:**
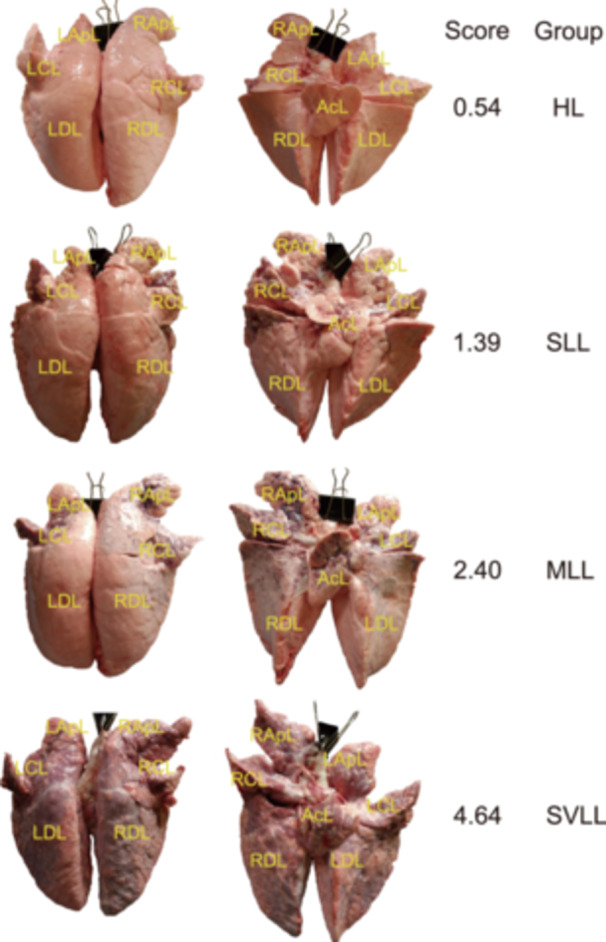
Lung lesion scoring for the grouping of 613 F_7_ pigs. The anterior and posterior views of the lung were photographed for each pig using a digital camera. Six sections of the anterior lung, including the left apical lobe (LApL), right apical lobe (RApL), left cardiac lobe (LCL), right cardiac lobe (RCL), left diaphragmatic lobe (LDL), and right diaphragmatic lobe (RDL), and seven sections of the posterior lobe, including the LApL, RApL, LCL, RCL, LDL, RDL, and accessory lobe (AcL), were scored using previously described methods (Zhang et al. [20] and Methods). Pigs with a final lung lesion score ranging from 0 to 0.75 were classified into the healthy lung group (HL, *n* = 51), those with a final score ranging from 0.75 to 1.50 were classified into the slight lung lesion group (SLL, *n* = 217), those with a final score ranging from 1.5 to 3.00 were classified into the moderate lung lesion group (MLL, *n* = 218), and those with a final score >3 were classified into the severe lung lesion group (SVLL, *n* = 127).

**Figure 4:**
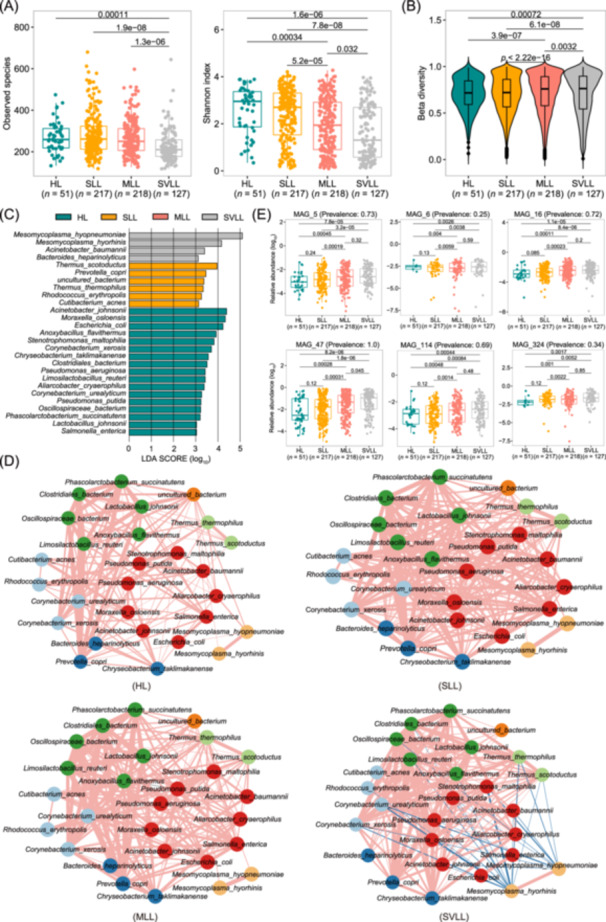
Identifying bacterial taxa and potential functional capacities associated with lung lesions. (A and B) Comparison of the *α*‐diversity (observed species and Shannon indices) and *β*‐diversity of the lung microbiome among four pig groups with different severities of lung lesions in the F_7_ population: The healthy lung (HL, dark green), slight lung lesion (SLL, orange), moderate lung lesion (MLL, light red), and severe lung lesion (SVLL, light gray) groups. The *β*‐diversity was calculated based on pairwise Bray–Curtis distances. Wilcoxon tests were used for the comparison of the *α*‐ and *β*‐diversity values across the four groups. The box lengths indicate the interquartile range. The centerline shows the median, the whiskers indicate the lowest and highest values within 1.5 times the interquartile range from the first to third quartiles, and the points indicate outliers from the whiskers. (C) The core species enriched in each pig group according to linear discriminant analysis effect size (LEfSe) analysis. (D) Co‐abundance network of 27 differential bacterial species. Interactions between species (nodes) are represented by connecting lines (edges), and each node is colored according to the phylum to which it belongs. The colors of the edge lines represent positive (light red) or negative (dark blue) interactions, and the width of the edge represents the magnitude of the Spearman correlation coefficient between species. (E) Significant associations of *M. hyopneumoniae* MAGs with lung lesions. Wilcoxon tests were used for the comparison of relative abundances. The legends for the box plots are the same as those for (A and B).

To minimize the influence of various environmental factors on the experimental pigs, F_7_ pigs from the mosaic population used in this study were raised on the same farm under uniform conditions with the same commercial feed. This provided an opportunity to better understand the relationship of the lung microbiome with lung lesions using the PRGC90 and MAG catalogs. BAL fluid samples from 613 F_7_ pigs in the mosaic population were divided into four groups according to their lung lesion scores (Figure 3): the healthy lung (HL) (*n* = 51), slight lung lesion (SLL) (*n* = 217), moderate lung lesion (MLL) (*n* = 218), and severe lung lesion (SVLL) (*n* = 127) groups (see Methods). We first compared the *α* diversity and *β* diversity of the lung microbiota across the four groups. The HL group exhibited the highest *α* diversity based on the Shannon and observed species indices, and the *α* diversity decreased with increasing severity of lung lesions (Figure 4A). The *β*‐diversity also differed significantly among the four groups. The dissimilarity of the lung microbial compositions increased with the severity of the lung lesions (Figure 4B). Thus, the altered diversity of lung microbial compositions among the groups suggested an association between the lung microbiota and lung lesions.

We then identified the microbial species associated with lung lesions. A total of 27 bacterial species had significantly different abundances among the four pig groups (Figure 4C). The relative abundances of *M. hyopneumoniae* and *Mesomycoplasma hyorhinis* clearly increased with increasing severity of lung lesions (Figure S7A). In particular, *M. hyopneumoniae* showed the most significant association with pig lung lesions (LDA = 5.1, *p* = 1.3 × 10^−6^; Figure 4C). We further constructed a co‐occurrence network to determine the relationships among the 27 differential species described above (Figure 4D). Overall, the co‐occurrence network could be clearly divided into two submodules. One module contained only *M. hyopneumoniae* and *M. hyorhinis* and showed few connections with the other 25 differentially abundant species in the HL, SLL, and MLL groups (only several positive connections in the HL group), while 25 differentially abundant species in the other module were positively connected with each other. We observed two significant signatures of the interaction network among the four pig groups: (1) there were significantly fewer connections among 27 differential bacterial species in the HL group than in the other three groups (average number of edges per node: 8.6 vs. 13.9, *p* < 0.01), suggesting fewer interactions among bacterial species in healthy pigs. (2) Significantly negative interactions were detected between two *Mesomycoplasma* spp. and 14 differential species in the other module in pigs with the SVLL group (Figure 4D). On the basis of the most significant associations of two *Mesomycoplasma* spp. with lung lesions, decreased *α* diversity, and interaction networks (Figure 4 and Figure S7A), we speculated that an excessive abundance of *Mesomycoplasma* spp., especially *M. hyopneumoniae*, might decrease the diversity of other non‐*Mesomycoplasma* species and that *M. hyopneumoniae* is the key species associated with pig lung lesions.

The construction of MAGs in the pig lower respiratory tract microbiome allowed us to identify bacterial strains related to lung lesions. We focused on the MAGs belonging to *M. hyopneumoniae* and *M. hyorhinis*. Seven out of the 356 nonredundant MAGs identified in this study belonged to *M. hyopneumoniae*. Except for MAG_2 (Figure S7B), the other six *M. hyopneumoniae* MAGs showed significant associations with lung lesions. Compared to those in the HL group, the SVLL and MLL groups had significantly greater abundances of these six MAGs (*p* = 2.6 × 10^−3^ ~ 1.8 × 10^−6^) (Figure 4E). Different significance levels were detected for the *M. hyopneumoniae* MAGs, suggesting differences in pathogenicity among the strains. MAG_47 showed the most significant association, and its abundance increased with the severity of lung lesions (Figure 4E). Notably, the low prevalence (10.0%) is likely the reason that the association of MAG_2 did not reach significance. Only one MAG was identified for *M. hyorhinis*, and it was significantly enriched in pigs with SVLLs (Figure S7C).

We then tested whether other pathogens were responsible for the observed lung lesions in the experimental pigs. Given that metagenomic sequencing data can be used to identify bacterial pathogens and DNA viruses through sequence alignment, we downloaded a total of 242 complete genomes of *Actinobacillus pleuropneumoniae*, *Bordetella bronchiseptica*, *Porcine circovirus*, and *Suid alphaherpesvirus 1* from NCBI Refseq database. These pathogens have been previously reported to be associated with lung diseases in pigs. The metagenomic sequencing reads were aligned to these pathogen genomes, and the results showed no evidence of infection by these pathogens in the pig lower respiratory tract. Since porcine reproductive and respiratory syndrome virus (PRRSV) can also cause lung disease and lesion, we tested the PRRSV antibody levels in F7 pigs. Nearly all of the tested pigs (99.2%) showed positive for PRRSV antibody. We then compared the concentrations of PRRSV antibody levels among HL, SLL, MLL, and SVLL pig groups. However, no statistically significant differences were observed in the PRRSV antibody levels between these groups (*p *= 0.16) (Figure S8). We thought that positivity for PRRSV antibody should be attributable to the PRRSV vaccination (see Methods). Additionally, all experimental pigs in the F7 population had detailed feeding records, and there were no records of diseases, such as reproductive and respiratory syndrome. Thus, all these indicated that the lung lesions were not caused by the infection of other pathogens.

To further confirm the roles of *M. hyopneumoniae* strains in lung lesions across pig populations, we performed association analysis in another population of Berkshire × Licha cross lines containing 11 healthy pigs and 17 pigs with lung lesions (Methods). Interestingly, only two *Mesomycoplasma* spp. were significantly enriched in pigs with lung lesions, with *M. hyopneumoniae* showing the most significant association (LDA = 5.2, *p* = 3.0 × 10^−3^). Furthermore, eight out of 19 bacterial species enriched in healthy pigs were also detected in the F_7_ population (Figure S7D). Six out of seven *M. hyopneumoniae* MAGs were significantly associated with lung lesions, with *p* values ranging from 0.029 to 9.10 × 10^−4^ (Figure S7E). Again, MAG_47 exhibited the most significant association (*p* = 9.10 × 10^−4^). All these results further confirmed the significant association of *M. hyopneumoniae* strains with pig lung lesions. Strangely, the associations of *M. hyorhinis* and its MAGs with lung lesions did not reach statistical significance in the Berkshire × Licha cross lines (Figure S7F).

VFs play important roles in *M. hyopneumoniae* infection and pathogenesis [9]. We then investigated the associations of VFGs in the lower respiratory tract microbiome with the severity of lung lesions. A total of 28 lung lesion‐associated VF types were identified in the F_7_ population (Figure S9A). Sixteen out of these 28 VF types were significantly enriched in the SVLL group, including the P97/P102 paralogue family, P146, P102, P65, P159, and P216, which are known as adhesins that are involved in the adhesion of *M. hyopneumoniae* to host respiratory ciliated epithelial cells. The P97/P102 paralog family showed the most significant association (LDA = 3.8, *p* = 1.7 × 10^−7^) (Figure S9A). There were also nine VF types significantly enriched in the HL group. The host bacterial species of these nine VF types were negatively correlated with two *Mesomycoplasma* spp. in the SVLL group (Figure 4D and Table S9), suggesting that the enrichment of VF types in the HL group may have been caused by the high abundances of the host bacterial species.

The lung lesion‐associated VF types identified in the Yorkshire × Licha lines were highly similar to those detected in the F_7_ population (Figure S9B). The P97/P102 paralog family, P102, P65, P146, P159, and P216 were significantly enriched in pigs with lung lesions, and the P97/P102 paralog family had the most significant association. These results strongly indicated that the adhesion of *M. hyopneumoniae* strains mediated by VFs to host respiratory ciliated epithelial cells may play a vital role in lung lesions.

### Pangenomes and the distribution of VFs in *M. hyopneumoniae* strains

**Figure 5:**
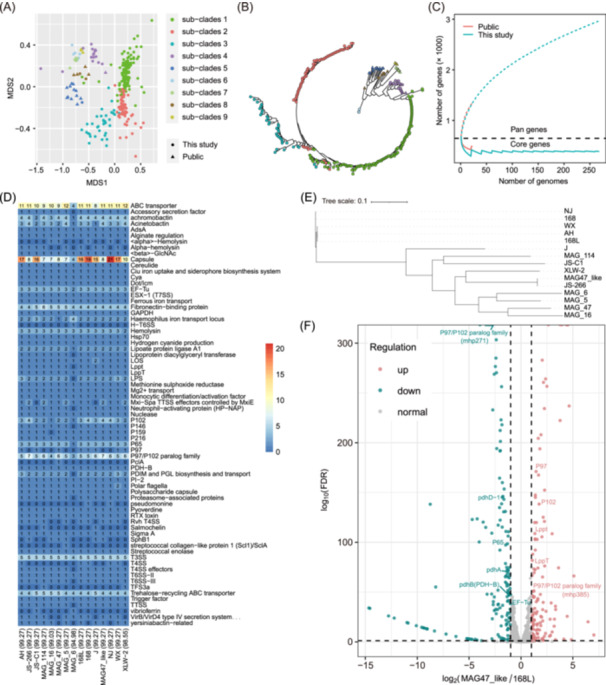
Pangenome and the distribution of virulence factors in *Mesomycoplasma hyopneumoniae* strains. (A) Classic multidimensional scaling (MDS) analysis of the 291 *M. hyopneumoniae* genomes based on the average nucleotide identity (ANI) distance matrix. Each dot represents a MAG, the stars indicate genomes of the isolates, and the triangles represent publicly available reference genomes of *M. hyopneumoniae*. Different colors indicate genetic subclades (subclades 1–9) inferred by rhierBAPs. (B) Maximum likelihood phylogenetic tree of all 291 *M. hyopneumoniae* genomes. The colors and shapes of the trees are consistent with those in (A). (C) Accumulation curves for pan‐genes and core genes in 262 *M. hyopneumoniae* MAGs reconstructed in this study, six genomes of isolates cultured from different pig populations, and 23 *M. hyopneumoniae* genomes downloaded from the NCBI RefSeq database. (D) The compositions and gene numbers of virulence factors (VFs) in 15 *M. hyopneumoniae* genomes, including five nonredundant MAGs and 10 genomes of the isolates. The virulence factor types are marked on the right. The numbers in the grids and color gradient indicate the gene numbers of each VF type in 15 strains. The percentage in brackets near the MAG name indicates completeness. (E) Phylogenetic tree of 15 *M. hyopneumoniae* genomes based on core genes. (F) Differentially expressed genes between the MAG47_like and 168L strains in pure culture. The x‐axis indicates log_2_ (fold change), and the y‐axis shows log_10_ (false discovery rate [FDR]). FDR‐corrected two‐sided *p* values were calculated with DeSeq 2 in the R package.

Among the 1965 MAGs (before dereplication) constructed in this study, the largest number of MAGs was obtained for *M. hyopneumoniae* (*n* = 262). A total of 291 *M. hyopneumoniae* genomes were used for phylogenetic and pangenome analyses, including 262 MAGs constructed in this study, six genomes from isolates cultured from different pig populations, and 23 *M. hyopneumoniae* genomes downloaded from the NCBI RefSeq database (Table S10). Nine subclades were obtained by Bayesian clustering based on multiple sequence alignments of 393 core genes (present in >95.0% of the genomes). All 23 *M. hyopneumoniae* genomes downloaded from the NCBI RefSeq database were present in five out of nine clusters, and all showed greater genetic similarity (closer classical multidimensional scaling distance) than the MAGs reconstructed in this study (Figure 5A). There were five clusters containing the retrieved MAGs that exhibited high genetic distance. A maximum‐likelihood phylogenetic tree was constructed based on multiple sequence alignments generated from 393 core genes and revealed that the genomes from the public database spanned a limited phylogenetic space, whereas the MAGs exhibited a broad phylogenetic structure (Figure 5B). Therefore, the reconstructed MAGs greatly expanded the genetic diversity of *M. hyopneumoniae* genomes.

In addition to 393 core genes, a total of 3136 pan‐genes were obtained from the 291 *M. hyopneumoniae* genomes via the Roary pipeline. Among them, 2373 (76.0%) genes were detected in fewer than 44 (15.0%) genomes, indicating a strain‐specific gene composition. The number of pan‐genes in the *M. hyopneumoniae* genome did not tend to saturation, suggesting an increase in the repertoire of genes (Figure 5C). The reconstructed MAGs had a much greater size of collective pan‐genes than genomes downloaded from the database, further suggesting expanded genetic diversity of *M. hyopneumoniae* strains in this study. Based on the annotations of cluster of orthologous groups and KEGG pathways, there were no significant differences in the functional categories between the pan‐genes and core genes (Figure S10).

We then focused on the distribution of VF types in *M. hyopneumoniae* strains with all seven nonredundant *M. hyopneumoniae* MAGs and 10 complete genomes of *M. hyopneumoniae* isolates. Because the completeness of the two *M. hyopneumoniae* MAGs was <90.0%, a total of 15 genomes were ultimately used for analysis. As shown in Figure 5D, a total of 71 VF types were identified in these 15 strains. There was no significant difference in the composition of VF types among strains. Almost all 15 genomes carried high numbers of VFGs encoding adhesins and adhesion‐related proteins (such as the P97/P102 paralog family and fibronectin‐binding protein), ABC transporter, and capsule proteins (Figure 5D). To further compare the transcriptional conditions of these VFGs between distinct strains, two *M. hyopneumoniae* strains were chosen for RNA sequencing analysis, including one strain named MAG47_like, which was closest to lung lesion‐associated *M. hyopneumoniae* MAGs, and the other strain named 168L, which was distant from lung lesion‐associated MAGs, in the phylogenetic trees constructed with whole‐genome sequences (Figure 5E). Interestingly, compared with the 168L strain, the MAG47_like strain expressed higher levels of VFGs encoding adhesins and adhesion‐related proteins, including the P97/P102 paralog family, P102, P97, LppT, and Lppt, but exhibited lower expression levels of *pdhB*, *pdhA*, *pdhD‐1*, and *EF‐Tu*, which encode *M. hyopneumoniae* surface‐displayed proteins that can bind complement factor H and result in the inhibition of the host complement pathway related to immune evasion [21] (Figure 5F). This result suggested that the MAG47_like strain could more strongly adhere to host bronchial ciliated epithelial cells than the 168L strain. Taken together, the results indicate that although the pangenomes and the composition of VFs were not significantly different among the different *M. hyopneumoniae* strains, differential expression levels of adhesion‐ and immunomodulation‐related VFs likely determine the adhesion and pathogenicity abilities of *M. hyopneumoniae* strains.

### Elucidating the mechanism of *M. hyopneumoniae* causing pig lung lesions via in vitro infection of bronchial ciliated epithelial cells and gene expression analysis

**Figure 6:**
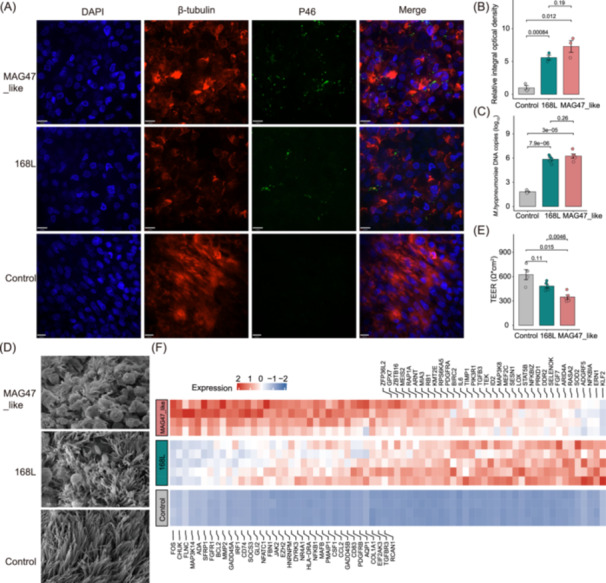
Elucidating the mechanism of *Mesomycoplasma hyopneumoniae* causing pig lung lesions via in vitro infection of bronchial ciliated epithelial cells (BECs) and gene expression analysis. (A) Adhesion of *M. hyopneumoniae* strains to BECs. Confocal immunofluorescence microscopy images show pig BECs infected with two *M. hyopneumoniae* strains at 24 hpi at the air–liquid interface. The cell nucleus, cilia, and *M. hyopneumoniae* cells were stained with DAPI (4′,6‐diamidino‐2‐phenylindole) (blue), β‐tubulin (red), and P46 (green), respectively. (B) Comparison of the integral optical density (IOD) values of *M. hyopneumoniae* staining among controls (*n* = 3), MAG47_like‐infected cells (*n* = 3), and 168L‐infected cells (*n* = 3). The ratios of the IOD values are presented for infected cells relative to those for control cells. (C) Quantitative real‐time polymerase chain reaction (qPCR) confirmed the amount of *M. hyopneumoniae* cells that adhered to BECs. The error bars indicate the means ± SEMs and are representative of independent experiments. (D) *M. hyopneumoniae* infection damaged the cilia of BECs, as observed by scanning electron microscopy. (E) Comparison of transepithelial electrical resistance among MAG47_like‐infected, 168L‐infected and control cells. (F) Heatmap showing the expression levels of the differentially expressed genes (DEGs) enriched in the immune and inflammation response, oxidative stress, and cell apoptosis pathways in control (*n* = 4), MAG47_like‐infected (*n* = 4), and 168L‐infected (*n* = 5) BECs at 24 hpi. All illustrated changes are presented relative to controls (uninfected BECs). The color gradient from red to blue indicates the normalized expression levels of DEGs in the tested samples.

To confirm the role of *M. hyopneumoniae* strains in pig lung lesions and diseases, primary BECs were isolated from Large White piglets at age 35 days. The MAG47_like and 168L strains described above were used for in vitro infection experiments. After primary BECs developed into mature cilia and multilayer structures in an air‒liquid interface culture system (15 days) (see Methods), pig BECs were separately infected with two selected *M. hyopneumoniae* strains from the apical side. At 24 h post infection (24 hpi), immunostaining and quantitative real‐time polymerase chain reaction (qPCR) were used to quantify the number of *M. hyopneumoniae* cells adhering to BECs. As shown in Figure 6A,B, both strains adhered to BEC cilia. Compared to the 168L strain, the MAG47_like strain, which was most closely related to lung lesion‐associated *M. hyopneumoniae* MAGs adhered more bacterial cells to BEC cilia. The same tendency was also observed in qPCR analysis, although the difference was not significant (*p *= 0.26, Figure 6C). This confirmed the different adhesion abilities of lung lesion‐associated *M. hyopneumoniae* strains on the cilia of BECs. Cilial damage was observed in *M. hyopneumoniae*‐infected BECs. Compared with MAG47_like strain‐infected cells in which most of the cilia were lost, 168L strain‐infected cells had a significantly smaller number of damaged cilia (Figure 6D). Significant disruption of the epithelial cell‒cell barrier was also demonstrated through a reduction in transepithelial electrical resistance (TEER) (Figure 6E). Compared with 168L strain‐infected BECs, MAG47_like strain‐infected BECs exhibited a significantly lower TEER (*p* = 4.6 × 10^−3^), suggesting more severe disruption of the epithelial cell‒cell barrier.

To further elucidate the mechanism by which *M. hyopneumoniae* strains adhere to the cilia of BECs, causing cilium loss and further causing lung lesions, RNA sequencing analysis was performed on four uninfected BEC samples (as controls), four mixtures of MAG47_like and infected BECs, and five mixtures of 168L and infected BECs at 24 hpi. An average of 14.5 Gb (from 11.8 to 19.0 Gb), 21.0 Gb (from 20.6 to 21.2 Gb), 21.2 Gb (from 21.1 to 21.2 Gb), 343.1 Mb (from 120.7 to 676.4 Mb), and 215.5 Mb (from 128.7 to 331.3 Mb) of clean sequence reads were obtained for the BEC controls, 168L‐infected BECs, MAG47_like‐infected BECs, MAG47_like strain, and 168L strain, respectively. Differentially expressed genes (DEGs) between infected cells and controls and between MAG47_like and 168L were analyzed. We first focused on DEGs between MAG47_like and 168L at 24 hpi because they exhibited differences in adhesion and cilial damage (described above). Compared with those in 168L, a total of 179 genes had higher expression levels, while 200 genes had lower expression levels, in MAG47_like (Figure S11 and Table S11). Genes encoding adhesins and adhesion‐related proteins (e.g., P97/P102 paralog family, P97, P102, and P159), *M. hyopneumoniae* surface‐displayed proteins related to evasion of host cell immune responses (PdhA, PdhB, PdhC, and GAPDH), and proteins related to protein secretion (Sec D and Sec G) exhibited significantly higher expression levels in MAG47_like than in 168L (Figure S11A). This suggested higher adhesion and stronger immune evasion abilities of MAG47_like in infected BECs than of 168L, which was consistent with the observation of a greater number of adhered MAG47_like cells in the cilia of BECs and the stronger pathogenicity of the MAG47_like strain. Notably, compared with those in pure cultures, genes encoding adhesion‐related proteins were significantly downregulated in bacterial cells infecting BECs for both strains at 24 hpi. However, the genes encoding immune evasion‐related proteins were significantly downregulated only in the 168L strain (Figure S11B,C).

Gene expression in BECs dramatically changed at 24 hpi. At the significance thresholds of fold change ≥2 and false discovery rate < 0.05, a total of 12,787 and 11,388 DEGs were identified between control cells and BECs infected with MAG47_like and 168L, respectively (Table S12). As we expected, the nuclear factor kappa‐light‐chain‐enhancer of activated B (NF‐κB), mitogen‐activated protein kinase (MAPK), cell apoptosis, T helper 1 and T helper 2 cell differentiation, tumor necrosis factor signaling, interleukin‐6 (IL6)/janus kinase 2 (JAK2)/signal transducer and activator of transcription signaling, and response to reactive oxygen species (ROS) pathways, which have been reported to be associated with cell damage caused by *M. hyopneumoniae* infection [9] were significantly enriched by DEGs (Table S13). For example, *MAP3K14*, *MAP3K8*, and *NFKB1*, which are involved in stimulating NF‐κB activity, and *FOS*, *SOCS3*, *IRF1*, *FLNC*, *HLA‐DRA*, and *PMAIP1*, which are associated with apoptosis and cell death [22], were upregulated by *M. hyopneumoniae* infection (Figure 6F). Notably, *NFKBIA* and *CHUK* (which inhibit the NF‐κB pathway), *SELENOK* (which plays a role in the protection of cells from ER stress‐induced apoptosis), and *BIRC2* and *BCL2* (which block apoptosis [23]) were also upregulated by *M. hyopneumoniae* infection (Figure 6F). A previous study demonstrated that *M. hyopneumoniae* infection could suppress the NF‐κB pathway and promote the survival of infected cells [24]. This antiapoptotic effect may allow bacterial adherence and colonization of host BECs in the initial stage of infection, which would subsequently cause a more severe proapoptotic effect, resulting in tissue damage [9]. The expression levels of *JAK2* and *IL6* (which are related to the immune response and inflammation), *SOD2* (which is associated with oxidative stress), and *GADD45A* and *GADD45B* (which are involved in the NF‐κB, MAPK, and apoptosis pathways and are related to oxidative stress [25]) were significantly increased by *M. hyopneumoniae* infection (Figure 6F). These gene expression results suggested that *M. hyopneumoniae* infection promoted immune and inflammation response, oxidative stress, and apoptosis in host BECs, resulting in cell death (cilium loss) and lung tissue lesions. Interestingly, compared with 168L‐infected BECs, MAG47_like‐infected cells had higher expression levels of *FOS*, *SOCS3*, *MAP3K14*, *CD74, GADD45A*, and *IRF1* but lower expression levels of *NFKBIA* (which inhibits NF‐κB), *SOD2* (which reduces pro‐oxidation and ROS levels [26]), and *KLF2* (which maintains epithelial integrity [27]) (Figure 6F), suggesting a stronger immune and inflammation response, oxidative stress, and cell apoptosis by the MAG47_like strain. This finding was consistent with the more severe cilial damage and disruption of the epithelial cell‒cell barrier caused by MAG47_like strain infection.

Overall, *M. hyopneumoniae* infection damaged BEC cilia and disrupted the epithelial cell‒cell barrier by increasing the expression levels of genes related to immune and inflammation response, oxidative stress, and cell apoptosis. The differences in adhesion ability and pathogenicity between *M. hyopneumoniae* strains were likely caused by differences in the expression levels of bacterial genes related to adhesion and immune evasion, which resulted in differences in the expression levels of host genes related to inflammation response, oxidative stress, and cell apoptosis.

### Comparison of phylogenetic compositions of the lung microbiome between humans with *M. pneumoniae* pneumonia and pigs infected with *M. hyopneumoniae*

Due to the similar symptoms between *M. pneumoniae* infection‐related lung disease in humans and *M. hyopneumoniae* infection‐related lung disease in pigs, we first performed a comparative genomic analysis between *M. pneumoniae* and *M. hyopneumoniae*. This analysis was based on 79 complete genomes of *M. pneumoniae* downloaded from the NCBI RefSeq database and 17 complete genomes of *M. hyopneumoniae* used in this study. The results revealed a highly distinct genetic distance between the genomes of *M. pneumoniae* and *M. hyopneumoniae* (Figure S12A). Phylogenetic evolution analysis using TimeTree showed that *M. pneumoniae* and *M. hyopneumoniae* diverged approximately 1574 million years ago (confidence interval: 1500.0–1648.3 million years ago). We further investigated the distribution of VF types and KOs in *M. pneumoniae* and *M. hyopneumoniae* genomes (Figure S12B,C). Lots of VF types were identified in both *M. pneumoniae* and *M. hyopneumoniae* genomes (54 and 72 VFs, respectively). Despite the distinct differences in the VF profiles, VFs related to the adhesion of pathogens to host cells, such as P97/P102 paralog family, were identified in both *M. hyopneumoniae* and *M. pneumoniae* genomes with high prevalence. A total of 293 KOs were shared between two species (Figure S12D), with these shared KOs being enriched in the pathways related to the ribosome, aminoacyl‐tRNA biosynthesis, and carbon metabolism (Figure S12E). Furthermore, two pathways of Protein export and Bacterial secretion system related to bacterial pathogenicity were also enriched with shared KOs. These findings suggested that although *M. hyopneumoniae* and *M. pneumoniae* are distinct pathogens in pigs and humans, respectively, they might share similar pathogenic mechanism in causing lung diseases.

We then compared the phylogenetic compositions and functional capacities of the lung microbiome between *M. pneumoniae*‐infected humans and *M. hyopneumoniae*‐infected pigs using metagenomic data generated in this study and 46 publicly available metagenomic sequencing data sets of BAL fluid from children with *M. pneumoniae* pneumonia [7]. At the phylum level, all 15 phyla identified in the human lung microbiome were included among the phyla detected in the pig lung microbiome, both having the following four predominant phyla (their relative abundances were among the top five): Proteobacteria, Bacteroidota, Firmicutes, and Tenericutes (Figure S13A). At the species level, a total of 90 bacterial species were identified in the human metagenomic data. Among them, 65 species were also present in the pig metagenome data (72.2%) (Figure S13B). Nine bacterial species were commonly identified among the top 20 species in terms of relative abundance in both the human and pig data sets (Figure S13C). At the functional capacity level, nearly all the KEGG pathways identified in the human lung microbiome (309/310) were also detected in the pig lung microbiome (Figure S13D). Seven KEGG pathways had high abundance in both the human and pig lung microbiomes (ranking among the top 20) (Figure S13E). All these results suggested that the lung microbiome compositions of humans with *M. pneumoniae* pneumonia were highly similar to those of pigs with *M. hyopneumoniae*‐caused lung lesions.

## DISCUSSION

To systematically investigate the role of the swine respiratory tract microbiome in respiratory diseases, we conducted large‐scale metagenomic sequencing of microbial samples from the swine lower respiratory tract and constructed microbial gene and MAG catalogs. We further elucidated the mechanism by which *M. hyopneumoniae* adhesion causes cilial damage and epithelial integrity disruption by performing in vitro infection of BECs, immunohistochemistry, VF analysis, and RNA sequencing. To our knowledge, this study provides the first microbial gene and MAG catalogs of the swine respiratory microbiome and has yielded new insights into the mechanism of action of different *M. hyopneumoniae* strains in lung diseases.

Although several studies have reported gene catalogs of the pig gut microbiota [28, 29], to our knowledge, there have been no such studies on the pig respiratory microbiome. For humans, Dai et al. [7] have constructed a respiratory microbial gene catalog containing 2.3 million genes using samples from healthy children and children infected with *M. pneumoniae*. There are even fewer studies on metagenomic sequencing analysis of the lower respiratory tract microbiota [30]. This is primarily due to the following reasons: (1) It is difficult to collect microbial samples from the lower respiratory tract because of its complex structure [31]. There is a high risk of contamination from the upper respiratory tract microbiota [32]. (2) Given the low biomass of the lower respiratory tract microbiota, it is difficult to obtain sufficient DNA for next‐generation sequencing [33]. More importantly, there is a high proportion (over 90.0%) of host DNA contamination. This results in only a small amount of valid microbial sequencing data being obtained [15]. In this study, tracheal lavage and BAL fluid samples were obtained by separating the lungs and trachea from the thoracic cavity of pigs after slaughter. This helped avoid contamination by the microbiota from the upper respiratory tract. Furthermore, blank and control samples were set at the same time when tested samples were collected and sequenced. The result showed that the contamination was unlikely to have a substantial influence on the observed microbiome profiles. An unprecedented number of microbial genes (more than 10.3 million) that exhibited high diversity and representation was obtained by greatly increasing the sequencing depth to an average of 70.3 Gb per sample (Figure S1A) and using a broad sample source.

Several studies have investigated the composition of the lung microbial community in pigs [11, 34, 35]. However, most of these studies were performed based on the 16S rRNA gene sequencing, which can only describe the microbial composition at the low‐resolution taxonomic level. Consistent with our previous report using 16S rRNA gene sequencing [11], *Prevotella, Streptococcus*, and *Mesomycoplasma* were the dominant genera in the lower respiratory tract microbiome. Ma et al. [35] performed a comparative analysis of the pulmonary microbiome in healthy and diseased pigs based on the 16S rRNA gene sequencing and found the distinct bacterial communities between healthy and diseased pigs. Similarly, Siqueira et al. [34] also reported that *M. hyopneumoniae*, *M. hyorhinis*, *Streptococcus suis*, *G. parasuis*, and *E. coli* were highly abundant in the lung microbiome of healthy pigs by using shotgun metagenomic sequencing. The composition of the lower respiratory tract microbiome is influenced by multiple factors, such as host genetics, age, environments, and feeds. For example, a previous study demonstrated that the environmental factors including soil, air, water, and feed should shape the swine lung microbial communities, with airborne bacteria contributing the largest proportion [36]. In this study, the distinct compositions of the lung microbiota among five pig populations were likely attributable to variations in environments, diet, and host genetics (Breeds). All F7 pigs were raised in an unformed farm setting with standard commercial feed under the same environmental conditions. Although pigs from the Berkshire × Licha cross line, Tibetan, and Erhualian pigs were also used in this study, these three pig breeds were raised in different farms with different diets. The combined influence of breed, farming environment, diet, and geographical location complicated the ability to distinguish the effect of each factor on the composition of the lower respiratory tract microbiome.

The construction of MAGs significantly increased the number of microbial genomes. This approach could be used to obtain strain‐specific genome information [37]. *M. hyopneumoniae*, which exhibited high abundance in pig lower respiratory tract microbiome, is a respiratory pathogen that can cause endemic pneumonia and cause considerable economic losses to pig farms [5]. A previous study revealed genetic variations in *M. hyopneumoniae* strains based on 16 selected genes [38], but whole‐genome surveys have not been carried out. Here, for the first time, we have reported the genetic diversity of *M. hyopneumoniae* strains in the porcine respiratory microbiome. Consistent with reports in which the functions of up to 30.0% of genes remained unknown in the current reference genomes [10], many pan‐genes could not be matched to COG categories and KEGG pathways. Furthermore, most of the pan‐genes were also strain specific. Compared with the 23 *M. hyopneumoniae* genomes available in the NCBI RefSeq database, the *M. hyopneumoniae* MAGs reconstructed in this study were more widely distributed in the phylogenetic tree (Figure 5B). These results revealed an extremely high degree of genetic variation in *M. hyopneumoniae* strains. We have provided large‐scale *M. hyopneumoniae* genome resources for facilitating the investigation of the roles of *M. hyopneumoniae* in respiratory diseases at the strain level.

The *α*‐diversity of the lung microbiome decreased with the severity of lung lesions. High microbial diversity has a favorable effect on maintaining homeostasis of the microecosystem, whereas low microbial diversity is associated with clinical features of pneumonia in the human lung microbiome [32]. The relative abundance of *M. hyopneumoniae* was negatively correlated with that of ten bacterial species in the SVLL group (Figure 4D). Consistent with this, a previous report indicated that *M. pneumoniae* can decrease the abundance of other bacteria by direct competition in the human respiratory microbiome [7]. Using 16S rRNA sequencing, we previously found that *Mesomycoplasma* was associated with lung lesions [11]. Here, the relative abundance of *M. hyopneumoniae* significantly increased with the severity of the lung lesions. Seven *M. hyopneumoniae* MAGs were associated with lung lesions in F_7_ pigs and the Berkshire × Licha cross lines at different significance levels. This result suggested the role of *M. hyopneumoniae* strains in lung lesions and revealed differences in pathogenicity among strains. *M. hyopneumoniae* is known as a pathogen that attaches to the cilia and surface of the epithelium to colonize the host and results in damage and loss of cilia and even epithelial cell death [5]. In vitro infection experiments in this study confirmed the effect of *M. hyopneumoniae* on the loss of BEC cilia and the disruption of the epithelial cell‒cell barrier, but the severity of these effects differed among strains (Figure 6). VFs aid pathogens in quickly adapting to shifts in the environment and improve the adhesion and invasion of pathogens to host cells [19]. They also help pathogens escape from the host's innate and adaptive immune systems [39]. A previous study demonstrated that adhesin proteins were associated with the pathogenicity of *M. hyopnemoniae* [40]. However, we did not observe a significant difference in the composition of VFs among the different strains. We indeed detected differential expression of VFGs encoding adhesins and genes related to immune evasion between strains with high (MAG47_like) and low (168 L) pathogenicity both in pure culture and at 24 hpi. Consistent with our observations, comparative genomics and proteomics also revealed that pathogenic and nonpathogenic strains of *M. hyopneumoniae* shared almost their entire repertoire of known adhesins, and one of the possible explanations for the difference in their adhesion capacities could be the difference in transcription level [40]. DEG analysis of BECs infected with *M. hyopneumoniae* strains with different levels of pathogenicity was performed by RNA sequencing. The increased expression levels of genes related to immune and inflammation response, oxidative stress, and cell apoptosis caused by *M. hyopneumoniae* infection were consistent with previous reports [9, 41] and suggested a mechanism by which *M. hyopneumoniae* causes cilial loss and disrupts epithelial integrity.

Both pigs infected *M. hyopneumoniae* and humans infected *M. pneumoniae* showed lung inflammation and disease. However, no evidence has been reported to indicate cross‐species infection between humans and pigs by these two bacterial pathogens. These two bacterial species belong to two different families and a highly distinct genetic distance was found between them. Despite this, we identified VFGs related to the adhesion of both pathogens to host cells in both *M. hyopneumoniae* and *M. pneumoniae* genomes with high prevalence. Additionally, a high percentage of shared KOs was observed. Combining the high similarity of phylogenetic compositions of the lung microbiome between humans with *M. pneumoniae* pneumonia and pigs infected with *M. hyopneumoniae*, the results suggested their similar pathogenic mechanisms causing lung diseases. These findings indicate that pigs may serve as the animal model for investigating the pathogenic mechanism of *M. pneumoniae* causing lung diseases in humans.

## CONCLUSION

In conclusion, we constructed a nonredundant gene catalog that contained an unprecedented number of 10,031,593 microbial genes (PRGC90) and recovered 356 MAGs from the swine lower respiratory tract microbiome. Using these catalogs, we found the heterogeneity in the microbial composition of the swine lower respiratory tract microbiome among individuals. We analyzed the pangenomes and investigated the distribution of VFs in *M. hyopneumoniae* strains. More importantly, we confirmed the effect of *M. hyopneumoniae* adhesion on the loss of BEC cilia and the disruption of epithelial integrity and elucidated the possible mechanism involved. However, several limitations existed in this study. First, although we conducted a high‐deep metagenomic sequencing, only 1.52 Gb clean data on average were obtained for each sample due to the high ratio of host DNA contamination. This let us have to perform co‐assembly analysis to obtain more high quality contigs, and also directly affected the completeness of predicted genes and constructed MAGs. Furthermore, the relatively small size of the clean sequence data also limited the capture of low‐abundant bacterial species. Thereby, it decreased the overall diversity of the pig lower respiratory tract microbiome. Second, we tried our best to avoid contaminations during the whole experimental procedure. However, it was far from sterile conditions that have been used in humans during surgery or bronchoscopy. The results from this study provided important resources for the research on the swine lower respiratory tract microbiome and could aid in the diagnosis and treatment of lung diseases.

## METHODS

### Experimental animals and sample collection

The subjects used in this study included 618 pigs from a mosaic population, 28 individuals of a Berkshire × Licha cross line, 11 Tibetan pigs, nine Erhualian pigs, and nine wild boars (Table S1). The mosaic population was constructed by intercrossing four Western breeds (Duroc, Landrace, Large White, and Pietrain) and four Chinese breeds (Bamaxiang, Erhualian, Laiwu, and Tibetan). All pigs were raised under uniform indoor conditions at the experimental farm in Nanchang, Jiangxi Province. Seven vaccines were administered to these experimental pigs, including classical swine fever and *P. circovirus* type II vaccines (Vaccination at 15 days of age), streptococcal disease and pseudorabies vaccines at the age of 24 days, foot‐and‐mouth vaccine at 32 days of age, PRRSV and *G. parasuis* vaccines at 40 days of age. A total of 687 samples were collected from 618 F_7_ pigs from this mosaic population at age 240 days with sterile PBS after slaughter, including 613 BAL fluid samples and 74 tracheal lavage fluid samples. Twenty‐eight pigs from a commercial Berkshire × Licha line raised on a commercial farm in Dingnan, Jiangxi Province, were slaughtered, and 28 BAL fluid samples were obtained. BAL fluid samples were also collected from 11 Tibetan pigs that were initially raised under semi‐free conditions in Ganzi, Sichuan Province; the pigs were subsequently transferred to a Nanchang farm and further raised for 1–3 months. BAL fluid samples from nine Erhualian pigs were also included in this study. These Erhualian pigs were raised on a farm located in Changzhou, Jiangsu Province. The similar vaccination procedure was also performed in Berkshire × Licha line, Tibetan, and Erhualian pigs. We collected nine BAL fluid samples from wild boars that were captured in the mountains of Jiangxi Province. All experimental pigs had recorded healthy status in detail during the whole‐feeding period and received no antibiotics or probiotics treatment for 2 months before sample collection. Several procedures were used to control possible contamination as much as possible: (1) Sterile PBS and 50‐mL sterile centrifuge tubes were separately prepared for each sample and aseptically and carefully stored. (2) After removal from the chest cavity, the lungs were placed in a clean place immediately for lavage. (3) All operators wore masks and disposable medical gloves. Sterile PBS was instilled into the lungs. After gently kneading the lungs for 2 min, we recycled liquids avoiding contact with any other materials. Approximately 45–50 mL of PBS lavage was collected from each lung within 30 min after slaughter. PBS from the same batch that underwent the same sampling process but was not used for lavage was chosen as a blank control. All samples were taken back to the laboratory on ice within 1 h. The PBS lavage mixture was centrifuged at 4000*g* for 30 min at 4°C in a laminar flow cabinet (Esco Life Sciences). The lavage pellet was subsequently transferred to a 2‐mL sterile tube and stored at –80°C until DNA extraction for subsequent analysis.

### Phenotype measurement

Porcine respiratory diseases are considered the most important health problems in pig production. However, the diagnosis of respiratory diseases in pigs is often challenging and relies on the combination of evaluating lung lesions via abattoir inspection. The degree of lung lesions can be treated as an important trait for assessing lung diseases in finishing pigs at slaughter [42]. Here, we adopted a scoring system to evaluate the lung lesions of experimental pigs at the slaughterhouse. In brief, the anterior and posterior views of each lung were photographed using a digital camera. Subsequently, the degree of lesions in each lung was scored using these photographs according to the methods and scoring criteria described in detailed in our previous study [20]. (I) The anterior lung was divided into six sections: the left and right apical lobe, left and right cardiac lobe, and left and right diaphragmatic lobe. The posterior lung was divided into seven sections: the left and right apical lobe, left and right cardiac lobe, left and right diaphragmatic lobe, and intermediate lobe (Figure 3). For each section, a score was assigned from zero to five, corresponding to the proportion of lesion area: 0%, 0%–20.0%, 20.0%–50.0%, 50.0%–75.0%, 75.0%–90.0%, and more than 90.0%. (II) Different sections were assigned different weights according to the area of each section. The weights of the left and right apical lobes, left and right cardiac lobes, left and right diaphragmatic lobes, and accessory lobe were 20.0% (10.0% for each of the left and right sections), 20.0%, 50.0%, and 10.0%, respectively. Finally, the summed score of the anterior lung × 50% + the summed score of six sections of the posterior lung corresponding to the anterior lung × 50.0% + accessory lobe score × 10.0% was treated as the final score of lung lesions for each experimental pig. Pigs with final scores ranging from 0 to 0.75 were classified into the HL group, those with scores ranging from 0.75 to 1.50 were classified into the SLL group, those with scores ranging from 1.5 to 3.00 were classified into the MLL group, and those with scores >3 were classified into the SVLL group. A total of six experienced panelists independently participated in the scoring. Then, three out of six scoring results with the highest pairwise correlation coefficient (>0.8) were used. The mean of the scores from these three panelists was treated as the lung lesion score of each animal. Rescoring had to be conducted if there were no three scoring results that met the requirements. A total of 675 experimental pigs were thoroughly phenotyped.

### Measurement of reproductive and respiratory syndrome virus (PRRSV) antibody

Blood samples were collected into a procoagulation tube during the bloodletting process. Serum was extracted from blood samples by centrifugation at 3000*g* for 30 min at 4°C. The IgG antibody of PRRSV in serum samples were measured using the PRRS X3 Ab ELIZA KIT (IDEXX) according to the manufacturer's instructions.

### Microbial DNA extraction and metagenomic sequencing

Microbial DNA was extracted from the lavage pellets using a QIAamp DNA Stool Mini Kit (Qiagen) according to the manufacturer's instructions. DNA extraction was carried out in a DNAse and UV‐treated laminar flow cabinet (Esco Life Sciences). The concentration and quality of the DNA samples were measured with a NanoDrop‐1000 and by 0.8% agarose gel electrophoresis. Metagenomic sequencing libraries were constructed using the VAHTS Universal DNA Library Prep Kit (Vazyme) following the manufacturer's instructions. In brief, DNA samples were randomly fragmented by sonication to a size of 300 bp. Then, the DNA fragments were end‐polished and A‐tailed at the 3′ end. Adaptors were ligated to the ends of the fragments for PCR amplification. After purification, all libraries were sequenced with the 150 bp paired‐end strategy on a DNBSEQ‐T7 platform (BGI). To investigate the possible contamination introduced during sample collection and sequencing, we collected six PBS samples from the same batch of PBS solution for lavage as blank controls and six samples of mixed reagents that were used for library construction and sequencing as sequencing background control samples. High‐throughput sequencing was performed for all 12 samples. Because the DNA concentrations obtained from the blank control samples were insufficient for library construction, these DNA samples were condensed to obtain sufficient material for library construction and sequencing.

### Metagenomic sequence assembly

The adapters and low‐quality sequences were filtered from the raw data using fastp (v0.20.1) [43]. Host genomic DNA sequence contamination was subsequently removed from sequencing data by mapping reads to the pig reference genome (Sscrofa11.1) using Bowtie2 (v2.3.5.1) [44] with default parameters. The co‐assembly of the 744 metagenomic sequencing data sets was performed by MEGAHIT (v1.2.9, ‐‐min‐contig‐len 500) [45]. Single‐sample assembly of the 744 metagenomic sequences was also performed using MEGAHIT [45] with the same parameters as those used for co‐assembly.

### Construction of the microbial gene catalog

Assembled contigs from both co‐assembly and single‐sample assembly were used for gene prediction by Prodigal (v2.6.3) [46] with the parameter “‐p meta.” Genes with lengths ≥100 bp were retained for further analyses, and genes with lengths <100 bp but with a complete ORF (with both start and stop codons) were also retained. All genes were clustered at the protein level using CD‐HIT (v4.8.1) [47] following UniRef guidelines at 100.0% (PRGC100), 90.0% (PRGC90), and 50.0% (PRGC50) amino acid sequence identity. Genes belonging to eukaryotes (except fungi and protists) were excluded from the gene catalog by aligning all genes to the UniProt TrEMBL database (https://www.uniprot.org/statistics/TrEMBL).

### Taxonomic and functional annotation of microbial genes

The taxonomic assignment of the microbial genes in the catalog was performed based on the amino acid sequences of the proteins using DIAMOND (v2.0.12.150) [48] against the UniProt TrEMBL protein database with the threshold e‐value ≤ 1 × 10^−5^. Proteins that were not aligned to the database were defined as unknown proteins. For genes with multiple records in the output of DIAMOND [48], the taxonomic classifications were determined based on the lowest common ancestor algorithms by BASTA (v1.4.1) [49] with the options of an alignment length >25 and shared by at least 60.0% of hits. Similarly, proteins that were not assigned to any taxon were defined as unknown taxa. The eggNOG orthologues and COG functional categories were annotated by aligning genes to the eggNOG database (v5.0) using eggNOG‐mapper (v2.6.1) [50]. The annotations of KEGG orthologues were obtained with KOBAS (v3.0.3) [51] software (‐t blastout:tab, ‐s ko). Genes in the catalog were aligned against the dbCAN2 database (HMMdb V10) to obtain CAZyme annotations using HMMER (v3.1b2) [52]. The protein amino acid sequences of genes were aligned to the VFDB [19] to obtain virulence factor annotations via BLAST (v2.12.0) [53]. The VFG sequences of the host bacteria were determined by aligning the gene sequences to both the VFDB and UniProt TrEMBL protein databases. If a gene sequence was simultaneously mapped to a VFG and a microbial taxon, we considered that microbial taxon as the host bacterium of this VFG. The VF types refer to a class of VFs encoded by the same kind of VFGs. The procedure for functional annotation of MAGs was the same as that for gene annotation. For all functional alignments of protein sequences, the annotated hit(s) with the highest scores were selected for subsequent analyses [54]. To compare the microbial composition of the lung microbiome between humans with *M. pneumoniae* pneumonia and *M. hyopneumoniae*‐infected pigs, we downloaded 46 publicly available metagenomic sequencing data sets of BAL fluid samples from children with *M. pneumoniae* pneumonia [7]. The procedures for raw data processing, assembly, construction of a nonredundant gene catalog, taxonomic annotation, and abundance calculation were similar to those used in this study.

### Metagenomic binning

Three binning software programs, MetaBAT2 (v2.15) [55], Maxbin2 (v2.2.7) [56], and CONCOCT (v1.0.0) [57], were used for binning assembled contigs from a single sample. Bin sets obtained from three programs were combined and refined with the Binning_refiner module of MetaWRAP (v1.3.2) [58]. The quality of the bins was evaluated using CheckM (v1.0.18) [59]. MAGs with ≥50.0% completeness and ≤10.0% contamination were retained for subsequent analyses. To improve the quality of the MAGs, metagenomic sequencing reads mapped to the MAGs were reassembled with metaSPAdes (v3.13.0) [60] using the Reassemble_bins module of MetaWRAP [58]. Considering the low sequencing depth after removal of host genomic DNA contamination, to retrieve more MAGs, a co‐binning strategy was used in this study. MetaBAT2 (v2.15), Maxbin2 (v2.2.7), and VAMB (v3.0.2) [61] were used for co‐binning with the procedures applying to single‐sample binning. All MAGs were dereplicated using dRep (v3.2.2) [62] and clustered at the threshold of 99.0% ANI at the strain level and 95.0% ANI for SGBs. Taxonomic annotation of MAGs was performed by GTDB‐Tk (v2.1.0) based on the Genome Taxonomy Database (GTDB (release 207), https://gtdb.ecogenomic.org/). Those SGBs containing at least one reference genome in the GTDB were considered as known SGBs, and SGBs that could not be matched to any reference genomes (<95.0% ANI with all reference genomes in the GTDB) were defined as uSGBs [63]. The genome annotations of MAGs were performed using Prokka (v1.13) [64] with default parameters, except for the MAGs classified into *Mesomycoplasma* and *Ureaplasma* that were annotated with the option “‐‐gcode 4,” because in most of the bacteria from *Mesomycoplasma* and *Ureaplasma*, the general UGA stop codon encodes tryptophan in the translation using genetic code 4.

### Estimation of the abundances of genes, taxa, functional terms, and MAGs

Clean reads of each sample were mapped to the gene catalog using BWA MEM2 (v2.2.1) [65]. SAMtools (v1.7) [66] was used for converting the samfiles to bamfiles and for sorting. The counts of successfully assigned reads were calculated by FeatureCounts (v2.0.1) [67]. The relative abundance of each gene in each sample was calculated by the following formula:

$$\text{Relative abundance }=\;\frac{N_{i}/L_{i}}{\sum_{i=1}^{n}N_{i}/L_{i}},$$

where for gene i, $N_{i}$ is the number of reads mapped to gene i, $L_{i}$ represents the sequence length of gene i, and n is the number of genes per sample. The relative abundances of taxa and functional categories were calculated by summing the relative abundances of genes assigned to each category. The abundance of MAGs in each sample was calculated by the Quant_bins module in metaWRAP (v1.3.2) [58].

### PacBio HiFi sequencing of *M. hyopneumoniae* strains

*M. hyopneumoniae* strains (AH, JS‐266, JS‐C1, NJ, WX, and XLW‐2) were obtained from the Veterinary Institute, Jiangsu Academy of Agricultural Sciences, and cultured in KM2 medium at 37°C. *M. hyopneumoniae* cells were harvested from the culture medium at the logarithmic growth stage. High‐molecular‐weight genomic DNA of six *M. hyopneumoniae* strains was extracted by the cetyltrimethylammonium bromide method followed by purification with the Grandomics Genomic Kit (Grandomics). the SMRTbell Prep Kit (v3.0) was used for the construction of the PacBio HiFi sequencing library. Libraries were sequenced on the PacBio Revio platform according to the manufacturer's instructions. CCS software (https://github.com/PacificBiosciences/ccs) was used to generate high‐precision HiFi reads with a quality greater than Q20 from the raw sequencing reads. A total of >1 Gb (1.8–3.9) of HIFI data was obtained for each sample. These HIFI reads were used for whole‐genome assembly using both metaFlye (v2.9.2‐b1786) (‐pacbio‐hifi ‐‐meta ‐‐scaffold) [68] and hifiasm_meta (v0.3‐r073) [69] with default parameters. The quality of the assembled genomes was evaluated by CheckM (v1.0.18) [59]. A complete genome with one contiguously circled contig was obtained for all six strains.

### Phylogenetic analysis

The phylogenetic tree of all MAGs was constructed by PhyloPhlAn (v3.0.60) [70] based on 400 universal marker proteins with the following parameters: “‐‐diversity low ‐‐min_num_marker 50.” The phylogenetic tree for the 291 *M. hyopneumoniae* genomes was built with PhyloPhlAn using 393 core genes (present in more than 95.0% of the genomes) that were determined by Roary (v3.12.0, ‐e ‐z ‐i 95 ‐cd 95 ‐t 4) [71]. The parameters were set as follows: “‐‐diversity low ‐‐trim greedy ‐‐remove_fragmentary_entries.” The phylogenetic trees for 356 MAGs and 291 *M. hyopneumoniae* genomes were visualized using iTOL (v6.5.2) [72] and GraPhlAn (v1.1.3) [73], respectively.

### Pangenome analysis of *M. hyopneumoniae*

A total of 262 MAGs were constructed in this study; six genomes from isolates cultured from different pig populations and 23 *M. hyopneumoniae* genomes downloaded from the NCBI RefSeq database were included in the pangenome analysis. The MAGs meeting the following standards were retained for further analysis: 0.7 Mbps < genome size < 1.1 Mbps (the sizes of 23 *M. hyopneumoniae* genomes downloaded from the NCBI RefSeq repository ranged from 0.8 to 1 Mbps), containing <500 contigs, >90.0% completeness, and <5.0% contamination. Finally, all 291 *M. hyopneumoniae* genomes passed quality control and were used for pangenome analysis. Prokka (v1.13, ‐‐gcode 4) [64] was then used to annotate these genomes. The annotated genomes were processed with the Roary pipeline [71]. The genes present in >95.0% of the genomes with a minimum of 95.0% identity were defined as core genes and were used for phylogenetic analyses. The subclade was defined using rhierbaps (v 1.1.3) package [74, 75] in the R (v4.1.2) based on the concatenated nucleotide core gene alignment that was produced by PhyloPhlAn. Pairwise ANI values between genomes were calculated using FastANI (v1.32) [76]. Classical multidimensional scaling analysis was conducted based on the pairwise ANI distance matrix using the cmdscale function in R.

### RNA sequencing of two *M. hyopneumoniae* strains from pure culture

The cultured cells of two *M. hyopneumoniae* strains (MAG47_like and 168L) were collected during the logarithmic growth phase. Total RNA was extracted from bacterial cells with an RNA‐Quick Purification Kit (UU‐bio Technology) according to the manufacturer's instructions. RNA samples with an RNA integrity number (RIN) ≥ 7, OD260/280 = 1.8–2.0, and OD260/230 ≥ 2.0 were used to construct libraries. Ribosomal RNA was removed using the TIANSeq rRNA Depletion Kit (bacteria) (TIANGEN). cDNA libraries were constructed according to the Optimal Dual‐mode mRNA Library Prep Kit (BGI). The libraries were sequenced on the MGISEQ‐2000 (BGI) platform with a 2 × 150 bp paired‐end strategy.

### In vitro infection experiments of two *M. hyopneumoniae* strains

Pig BECs were isolated from the bronchi of SPF piglets at 35 days of age as described in a previous report [77] and cultured in an air–liquid interface (ALI) culture system that mimics the native conditions of the airway epithelium in vivo and is comparable to native tissue [78, 79]. Cells were considered well differentiated when wavy cilia appeared on at least 80.0% of the ALI‐BEC surfaces. Differentiated ALI‐BECs were maintained without antibiotics for 1 day before infection, washed three times with PBS, and incubated for 4 h at 37°C to allow mucus accumulation to a depth close to that of intact airways. ALI‐BECs were inoculated on the apical side of the filter with approximately 10^7^ CCUs (multiplicity of infection [MOI] = 20) of *M. hyopneumoniae* strains (MAG47_like and 168L) in 100 µL of Hanks' balanced salt solution (HBSS). Cells inoculated with only HBSS were used as a negative control. *M. hyopneumoniae* strains and BECs were incubated together for 24 h in a humidified atmosphere of 5.0% CO_2_ at 37°C for infection. To quantify adherent bacteria, infected and control cells were washed three times with PBS to remove nonadherent bacterial cells.

### Immunofluorescence assay of *M. hyopneumoniae* adhesion

Dual‐immunofluorescence microscopy was used to detect adhered *M. hyopneumoniae* cells as described previously [80]. Bacterial cell membranes were blocked in 1.0% bovine serum albumin (BSA) for 30 min before incubation with primary and secondary antibodies. The primary antibodies used were a mouse antibody against *M. hyopneumoniae* (1:100 dilution) (produced by the Jiangsu Academy of Agricultural Sciences) and Alexa Fluor 488‐conjugated goat antimouse IgG (1:400 dilution) (Beyotime Biotech). Then, Cy3‐conjugated goat antimouse IgG (1:400 dilution) (Beyotime Biotech) was used as the secondary antibody. To detect *β*‐tubulin on the surfaces of bacterial cells, after blocking, the cells were washed with PBS and incubated with a primary antibody against *β*‐tubulin (1:200 dilution) (Novus Bio) in 1.0% BSA overnight at 2°C to 8°C. Then, the bacterial cells were washed and incubated for 1 h with Alexa Fluor 555‐conjugated goat antirabbit IgG (1:400 dilution) (Beyotime Biotech). All cells were further stained with 2,4‐diamidino‐2‐phenylindole (DAPI) (Beyotime Biotech) for 5 min and washed with PBS at room temperature. The cells were imaged using an UltraView VoX laser scanning confocal microscope (PerkinElmer).

### Determination of the amount of adhered *M. hyopneumoniae* cells by quantitative PCR

*M. hyopneumoniae* DNA was extracted from 0.2 mL of culture medium from the lower chambers of the ALI culture system using a TIANamp Bacteria DNA kit (TIANGEN) according to the manufacturer's instructions. The extracted DNA was quantified by quantitative real‐time PCR using the TaqMan system. The primers and TaqMan‐BHQ probes were designed based on the conserved sequence of the P97 gene of *M. hyopneumoniae* [81]. Quantitative PCR was performed in an Applied Biosystems 7500 real‐time PCR system using the following cycling parameters: 50°C for 2 min; 95°C for 10 min; and 40 cycles of 95°C for 15 s, and 60°C for 1 min.

### Determination of the damage to BEC cilia by scanning electron microscopy

Filter membranes with BECs were washed in PBS and fixed in 2.5% glutaraldehyde in 0.1 M phosphate buffer (pH = 7.4). The specimens were dehydrated with a graded acetone series and dried in hexamethyldisilazane solution (Sigma‒Aldrich). The dried specimens were coated with a thin layer of platinum using the ion beam coater of a precision etching and coating system (Gatan France) and then observed using a Zeiss Ultra Plus field emission gun scanning electron microscope (FEGSEM).

### Measurement of TEER

To assess the integrity of the epithelial cell barrier, TEER was measured using a Millicell‐ERS (Electrical Resistance System) (Millipore). At 24 hpi, 500 µL of medium was added apically to the inset to assess the TEER of each culture inset. Before measuring the TEER of each culture, an empty culture inset was used as a blank, and the TEER value was calculated as (measured value—background value).

### Dual RNA sequencing of *M. hyopneumoniae* strains and infected BECs

The isolated strains (MAG47_like and 168L) of *M. hyopneumoniae* were obtained from the Veterinary Institute, Jiangsu Academy of Agricultural Sciences, and propagated in established ALI culture systems to infect BECs for 24 h at an MOI of 20:1. Both uninfected and infected cells were harvested by incubating BECs at 24 hpi. Total RNA was extracted using the TRIzol method. Prokaryotic and eukaryotic rRNA was removed using the TIANSeq rRNA Depletion Kit (bacteria) and TIANSeq rRNA Depletion Kit (H/M/R) (TIANGEN). cDNA libraries were constructed with an Optimal Dual‐mode mRNA Library Prep Kit (BGI). In brief, rRNA‐free RNA samples were fragmented to 200–600 nt by adding Fragmentation Buffer. First‐strand cDNA synthesis was performed using Strand Specificity Reagent and First Strand Enzyme Mix (BGI). Synthesis of the second strand was then conducted by adding Second Strand Buffer (dUTP) and Second Strand Enzyme Master Mix (BGI). The 5′ sequencing adapter was ligated to the 3′ end of the antisense cDNA. The resulting cDNA was amplified using a BGI Plug‐In Adapter Kit (BGI). The constructed libraries were sequenced on a MGISEQ‐2000 (BGI, China) platform with a 2 × 150 bp paired‐end strategy.

The raw sequence reads were initially trimmed by removing low‐quality and adapter sequences with Fastp (v0.23.2) [43]. All rRNA reads were removed using SortMeRNA (v4.3.6) [82] with default settings. Host and bacterial reads were collected by mapping against the pig reference genome (Sscrofa11.1) and *M. hyopneumoniae* genomes (Accession Nos. GCF_021383865.1 and GCF_000400855.1) using Bowtie2 (v2.4.5) [44], respectively. We first mapped host sequence reads to the pig reference genome (Sscrofa11.1) using STAR (v2.7.11a) [83] and then converted the SAM format to BAM format and sorted the reads using SAMtools (v1.15.1) [66]. StringTie (v2.2.1) [84] was then used for transcript assembly based on the alignments. Gene expression was quantified for each sample using featureCounts (v2.0.1) [67]. For *M. hyopneumoniae* sequence reads, clean sequences were mapped to the reference genomic sequences of *M. hyopneumoniae* using Bowtie2 (v2.4.5) [44]. The mapped reads were then quantified for each sample using featureCounts (v2.0.1). DEGs were identified by the DESeq. 2 (v1.34.0) R package [85]. To identify KEGG pathways enriched by DEGs whose difference levels were listed in the top 500, we performed enrichment analysis using the Cytoscape plug‐in ClueGo [86].

### Statistical analysis

The accumulation curve of the number of predicted genes in the gene catalog was bootstrapped 10 times at each sampling interval. Asymptotic regressions were carried out using the SSasymp and nls functions in the R *stats* package. Linear discriminant analysis effect size analysis (v1.1.01) [87] was used to identify MAGs, VFs, and KEGG pathways showing different enrichments among pig groups with different severities of lung lesions in F_7_ pigs and between healthy pigs and pigs with lung lesions in the Berkshire × Licha cross line at the threshold |LDA| > 2. The *α*‐diversity indices, including the observed species and the Shannon index, and the *β*‐diversity metric of the lung microbial composition based on the Bray‒Curtis distance were calculated with the R vegan (v2.5‐7) package [88]. PCoA was also performed with the R vegan package using Bray–Curtis dissimilarities. Comparisons of the *α* diversity and lung microbial composition among four pig groups with different severities of lung lesions were carried out using Wilcoxon tests. The co‐occurrence network of 27 bacterial species in each pig group was constructed based on Spearman rank correlations of the relative abundances of the bacterial species. The *p* values were calculated based on 999 permutations. Only interactions between bacterial species with correlation coefficients >0.3 and *p* < 0.05 were present in the co‐occurrence network. The co‐occurrence networks were visualized and annotated in Cytoscape (v3.9.1) [89].

## AUTHOR CONTRIBUTIONS

**Jingquan Li**: Writing—original draft; formal analysis; data curation; visualization. **Fei Huang**: Data curation; methodology; investigation. **Yunyan Zhou**: Methodology; visualization; formal analysis. **Tao Huang**: Data curation. **Xinkai Tong**: Data curation. **Mingpeng Zhang**: Data curation. **Jiaqi Chen**: Data curation. **Zhou Zhang**: Data curation. **Huipeng Du**: Data curation. **Zifeng Liu**: Data curation. **Meng Zhou**: Data curation. **Yiwen Xiahou**: Data curation. **Huashui Ai**: Data curation; supervision; methodology. **Congying Chen**: Supervision; writing—review and editing; validation. **Lusheng Huang**: Project administration; conceptualization; resources; writing—review and editing; funding acquisition.

## CONFLICT OF INTEREST STATEMENT

The authors declare no conflicts of interest.

## ETHICS STATEMENT

The project was approved by Animal Care and Use Committee (ACUC) in Jiangxi Agricultural University (No. JXAULL‐2021‐01‐10).

## Supporting information


**Figure S1.** Metagenomic sequencing depths in 744 tested samples and blank control samples.
**Figure S2.** The phylogenetic compositions of virus, fungi, and archaea in the pig lower respiratory tract microbiome with the pig lower respiratory tract gene catalog 90 (PRGC90).
**Figure S3.** Quality assessment of 356 metagenome‐assembled genomes (MAGs).
**Figure S4.** Host bacteria of virulence factor genes (VFGs).
**Figure S5.** Comparison of the diversity and microbial compositions of the trachea and lung microbial community.
**Figure S6.** The top 20 microbial species in different populations based on the relative abundances.
**Figure S7.** Association between the lung microbiome and lung lesions.
**Figure S8.** The comparison of the porcine reproductive and respiratory syndrome virus (PRRSV) antibody levels in serum samples among healthy lung, slight lung lesion, moderate lung lesion, and severe lung lesion pigs.
**Figure S9.** Associations of potential functional capacities of the lung microbiome with lung lesions in the F_7_ population and the Berkshire × Licha cross lines.
**Figure S10.** Functional annotations of *Mesomycoplasma hyopneumoniae* pan‐genomes.
**Figure S11.** Identification of differentially expressed genes (DEGs) between MAG47_like and 168L strains at 24 hpi and between bacterial cells infecting host bronchial epithelial cells (BECs) and pure cultured bacterial cells.
**Figure S12.** Comparison of the genomic structures between *Mycoplasma pneumoniae* and *Mesomycoplasma hyopneumoniae*.
**Figure S13.** Comparison of microbial compositions and potential functional capacities of the lung microbiome between humans and pigs.

**Table S1.** The information of 744 metagenomic sequencing samples.
**Table S2.** Information summary of the samples used in this study.
**Table S3.** Relative abundance of the bacterial species whose abundances were ranked in the top ten in the background controls and its prevalence and average abundance in tested samples.
**Table S4.** Relative abundance of the bacterial species whose abundances were ranked in the top ten in the background controls and its distribution and abundance in tested samples.
**Table S5.** The number and percentage of genes in the PRGC90 annotated to each functional item.
**Table S6.** Summary of 356 metagenome‐assembled genomes (MAGs).
**Table S7.** Statistics of virulence factor categories identified in the PRGC90.
**Table S8.** Host bacteria of virulence factor genes (VFGs) and the numbers of VFGs they carried.
**Table S9.** Host bacteria of virulence factor types at the species level.
**Table S10.** Summary of 291 *Mesomycoplasma hyopneumoniae* genomes.
**Table S11.** Differentially expressed genes (DEGs) between MAG_47_like and 168L strains after infecting pig bronchial epithelial cells (24 hpi).
**Table S12.** Differentially expressed genes (DEGs) between infected and uninfected bronchial epithelial cells at 24 hpi.
**Table S13.** Go terms and KEGG pathways enriched by differentially expressed genes (DEGs) between infected and uninfected bronchial epithelial cells (BECs) (only 500 up‐regulated and 500 down‐regulated genes are shown).

## Data Availability

All data needed to evaluate the conclusions in the paper are present in the paper and/or the Supplementary Materials. All pig metagenomic sequencing data are available in the GSA database (http://ngdc.cncb.ac.cn/gsa/browse/CRA007668) under accession number CRA007668. RNA‐seq data of pig bronchial epithelial cells and *Mesomycoplasma hyopneumoniae* strains were deposited in the GSA database under accession number: CRA015647 and CRA015646, respectively. All nonredundant MAGs and microbial genes in the gene catalog were also deposited in the National Genomics Data Center under bioProject accession PRJCA010893 (http://ngdc.cncb.ac.cn/bioproject/browse/PRJCA010893) with accession numbers OMIX006069 and OMIX006125, respectively. The source data and codes are available at GitHub (http://github.com/Jingquan-Li/PRGC). Supplementary materials (figures, tables, graphical abstract, slides, videos, Chinese translated version, and update materials) may be found in the online DOI or iMeta Science http://www.imeta.science/.
