# Supplementary material for: Comprehensive lung microbial gene and genome catalogs assist the mechanism survey of Mesomycoplasma hyopneumoniae strains causing pig lung lesions
Source: Imeta. 2024 Dec 26;3(6):e258. doi: 10.1002/imt2.258 (PMC11683470; doi:10.1002/imt2.258)
Supplement: Supplementary file 1 — Figure S1. Metagenomic sequencing depths in 744 tested samples and blank control samples. Figure S2. The phylogenetic compositions of virus, fungi, and archaea in the pig lower respiratory tract microbiome with the pig lower respiratory tract gene catalog 90 (PRGC90). Figure S3. Quality assessment of 356 metagenome‐assembled genomes (MAGs). Figure S4. Host bacteria of virulence factor genes (VFGs). Figure S5. Comparison of the diversity and microbial compositions of the trachea and lung microbial community. Figure S6. The top 20 microbial species in different populations based on the relative abundances. Figure S7. Association between the lung microbiome and lung lesions. Figure S8. The comparison of the porcine reproductive and respiratory syndrome virus (PRRSV) antibody levels in serum samples among healthy lung, slight lung lesion, moderate lung lesion, and severe lung lesion pigs. Figure S9. Associations of potential functional capacities of the lung microbiome with lung lesions in the F7 population and the Berkshire × Licha cross lines. Figure S10. Functional annotations of Mesomycoplasma hyopneumoniae pan‐genomes. Figure S11. Identification of differentially expressed genes (DEGs) between MAG47_like and 168L strains at 24 hpi and between bacterial cells infecting host bronchial epithelial cells (BECs) and pure cultured bacterial cells. Figure S12. Comparison of the genomic structures between Mycoplasma pneumoniae and Mesomycoplasma hyopneumoniae. Figure S13. Comparison of microbial compositions and potential functional capacities of the lung microbiome between humans and pigs. [file IMT2-3-e258-s001.docx]

**Supporting information to**

**Comprehensive lung microbial gene and genome catalogs assist the mechanism survey of *Mesomycoplasma hyopneumoniae* strains causing pig lung lesions**

**Running title:** Lung microbial gene and genome catalogs reveal the mechanism of pig lung lesions

Jingquan Li^#^, Fei Huang^#^, Yunyan Zhou^#^, Tao Huang, Xinkai Tong, Mingpeng Zhang, Jiaqi Chen, Zhou Zhang, Huipeng Du, Zifeng Liu, Meng Zhou, Yiwen Xiahou, Huashui Ai^*^, Congying Chen^*^, Lusheng Huang^*^

National Key Lab for Swine genetic improvement and germplasm innovation, Jiangxi Agricultural University, Nanchang 330045, China

^#^These authors contributed equally: Jingquan Li, Fei Huang, Yunyan Zhou

^*^Correspondence: lushenghuang@hotmail.com (Lusheng Huang), chencongying@jxau.edu.cn (Congying Chen), and aihsh@hotmail.com (Huashui Ai)


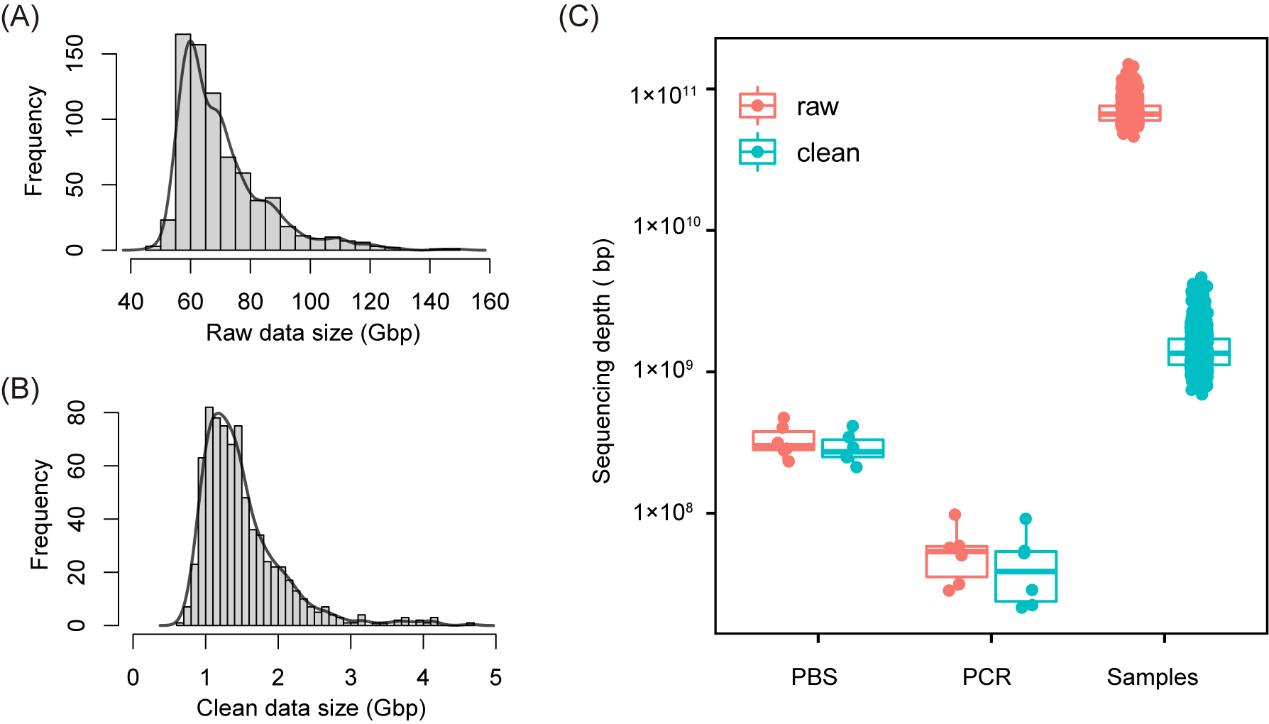


**Figure S1 Metagenomic sequencing depths in 744 tested samples and blank control samples.** (A)The distribution of raw data size. (B) the distribution of clean data sizes in 744 samples. The Kernel density curves are shown in the histograms. (C) Sequencing data size of blank control samples compared to that of tested samples. PBS, sterile phosphate-buffered saline (PBS) samples; PCR, mixed regents for library construction and sequencing. Sequencing depth of raw data and clean data for PBS, PCR, and tested samples.


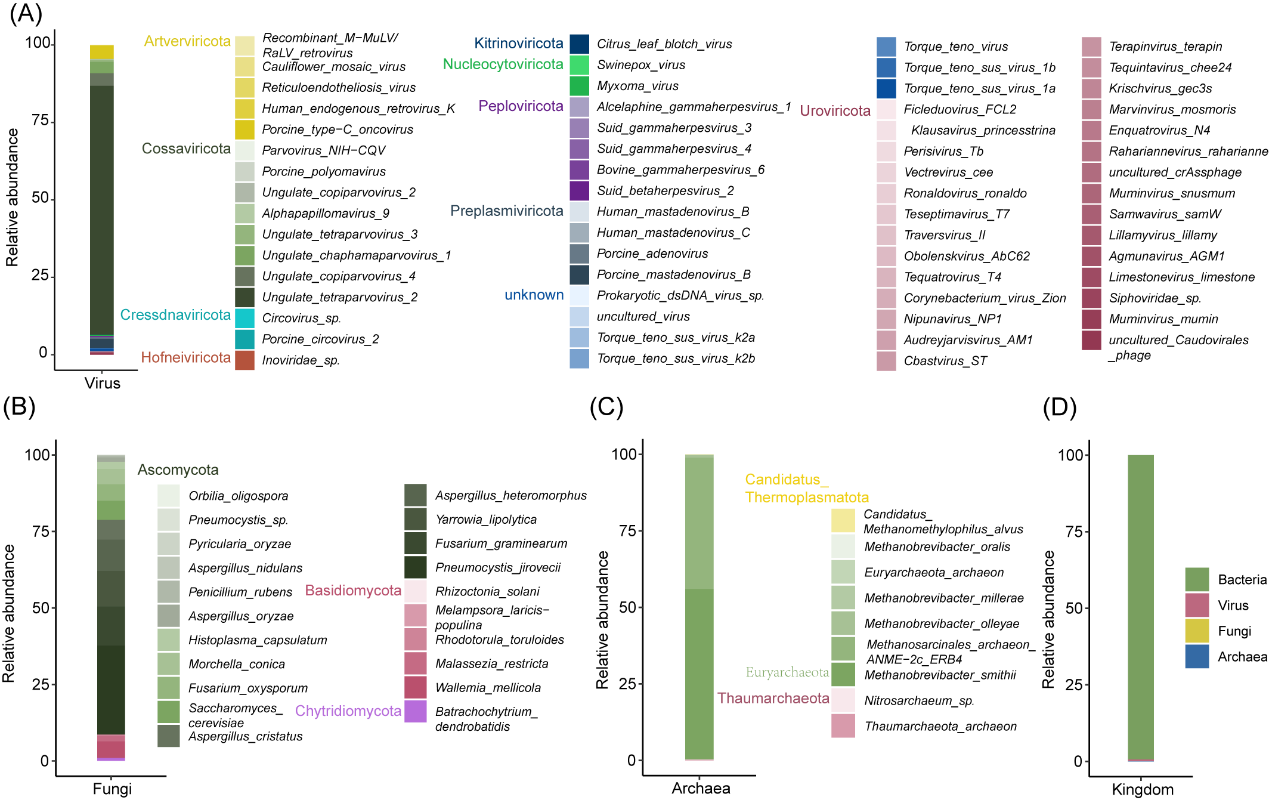


**Figure S2 The phylogenetic compositions of virus, fungi and archaea in the pig lower respiratory tract microbiome with the pig lower respiratory tract gene catalog 90 (PRGC90).** (A-C) The histograms showing the relative abundance of each microbial taxon in total abundance for virus (A), fungi (B), and archaea (C) in tested samples. Taxa are coloured by phylum and the species within each phylum. The names of the corresponding phyla and species are given in the keynote. (D) The relative abundance of bacteria, virus, fungi and archaea in tested samples.


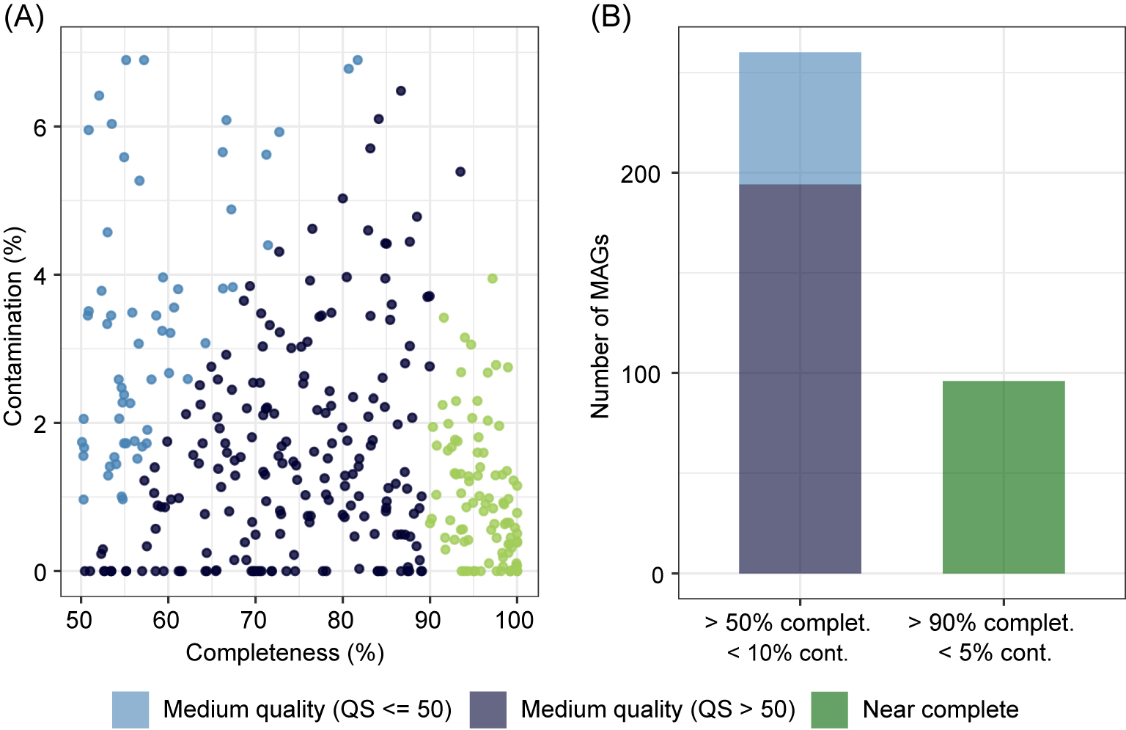


**Figure S3 Quality assessment of 356 metagenome-assembled genomes (MAGs).** (A) Distribution of the contamination and completeness rates of 356 MAGs estimated by CheckM. (B) The numbers of MAGs at each level of genome completeness and contamination. Quality score (QS) = completeness – (5 × contamination).


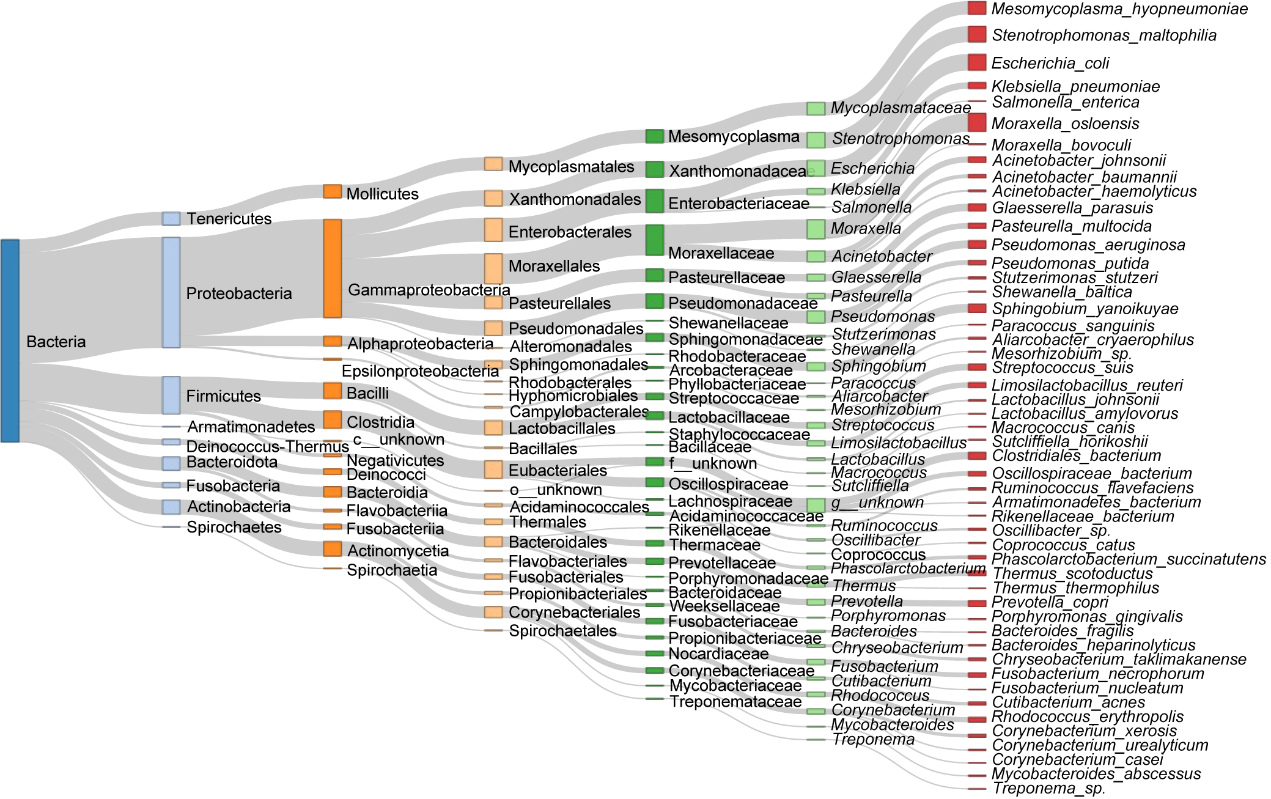


**Figure S4 Host bacteria of virulence factor genes (VFGs).** The distribution of host bacteria in which the numbers of harbored VFGs were ranked in the top 50. The colored rectangles from left to right represent different taxonomy levels from domain to species. The widths of the rectangles indicate the number of VFGs.


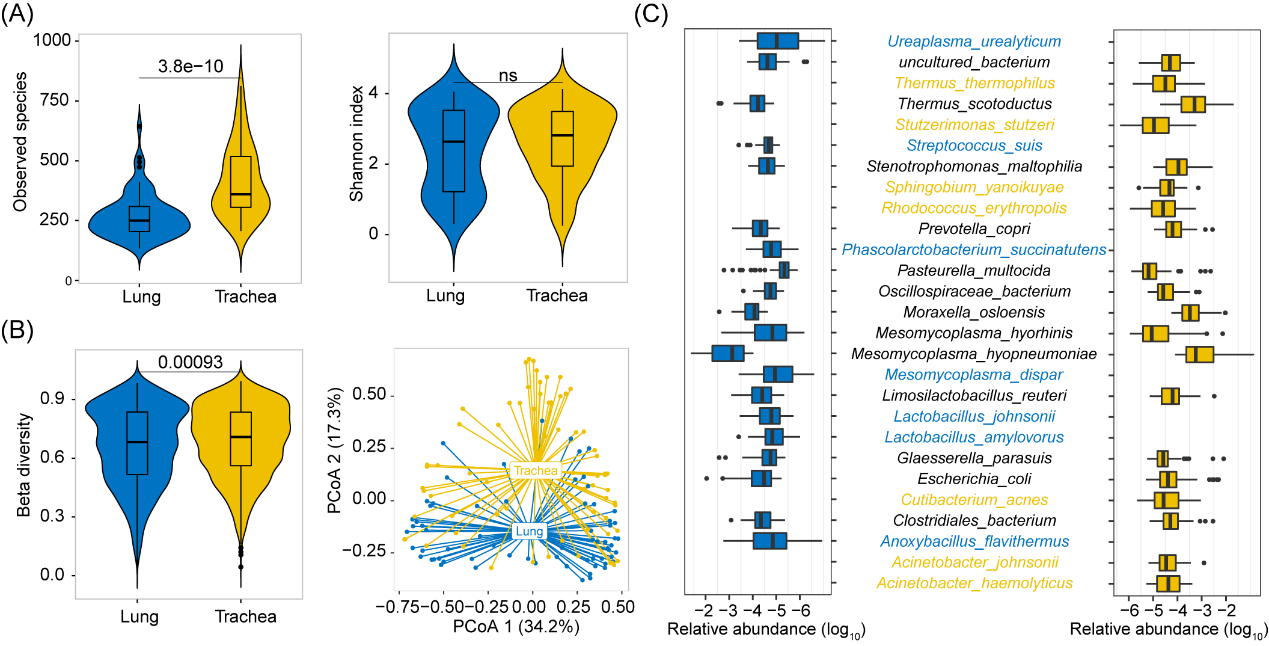


**Figure S5** **Comparison of the diversity and microbial compositions of the trachea and lung microbial community.** (A) Comparison of the *α*-diversity (observed species and Shannon index) of microbial compositions between lung and trachea samples. (B) Comparison of the *β*-diversity of microbial compositions between lung and trachea samples based on paired-wise Bray–Curtis distances. Principal coordinate analysis (PCoA) was performed based on the Bray–Curtis distances. (C) The top 20 species in relative abundances in the lung and trachea microbiome. The black color font indicates the top 20 species in both sample types, and the word colors corresponding to boxplot colors show the top 20 species specific to each sample type. The log_10_ (relative abundance) values are shown on the *X*-axis. *p* values of the comparison are present above the horizontal lines.


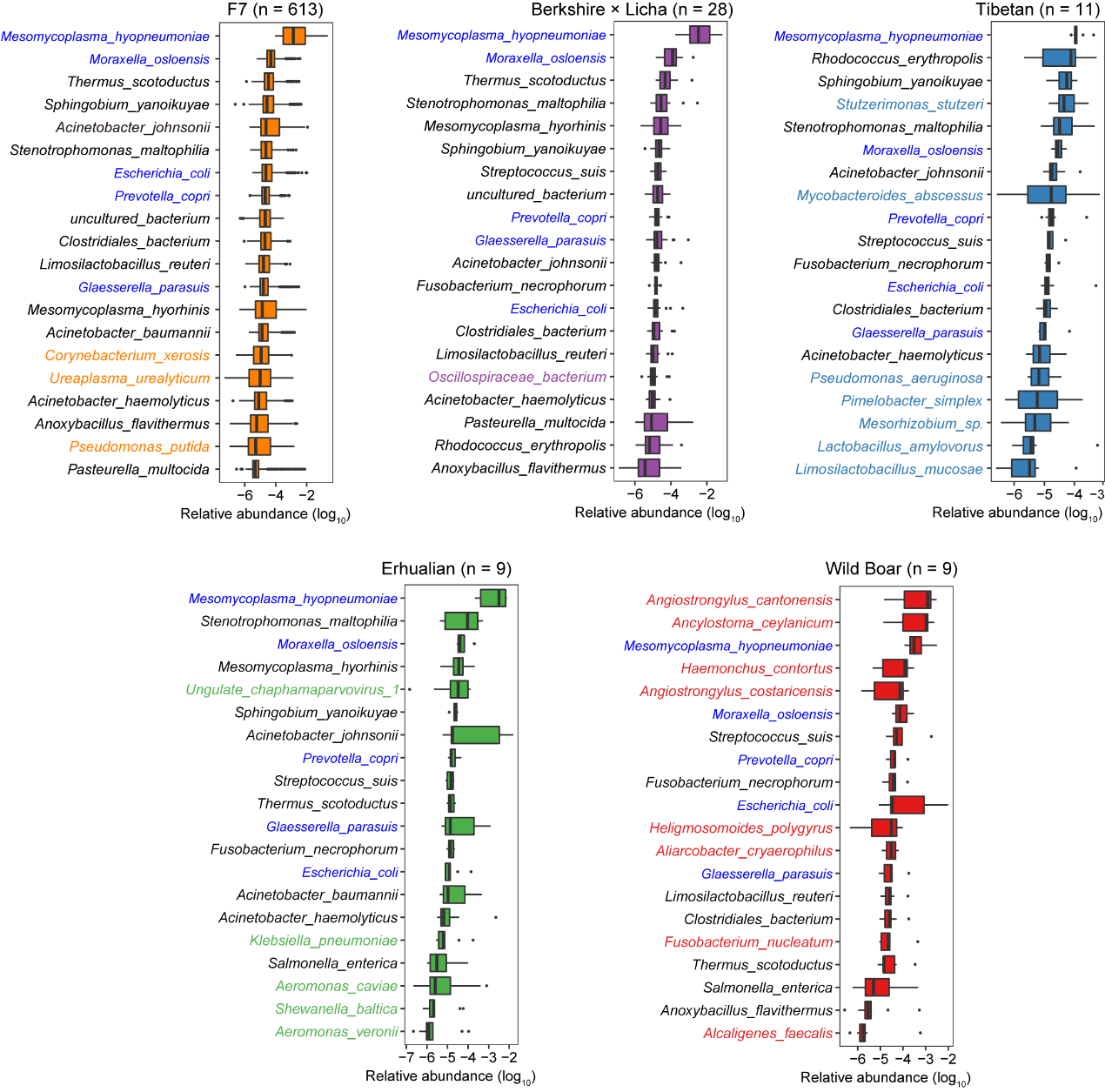


**Figure S6 The top 20 microbial species in different populations based on the relative abundances.** The top 20 species based on the relative abundances in the lower respiratory tract microbiome of F_7_, Berkshire × Licha line, Tibetan, Erhualian, and Wild boars. The core species were presented in ≥ 95% samples in each population. The species with blue texts were commonly identified in the lists of the top 20 in all five groups. Those species whose text colors correspond to the boxplots were specifically identified in that population. The box plots show the median (center line in the box), interquartile range (box limits), 1.5 times of interquartile range (whiskers), and outliers (dots).


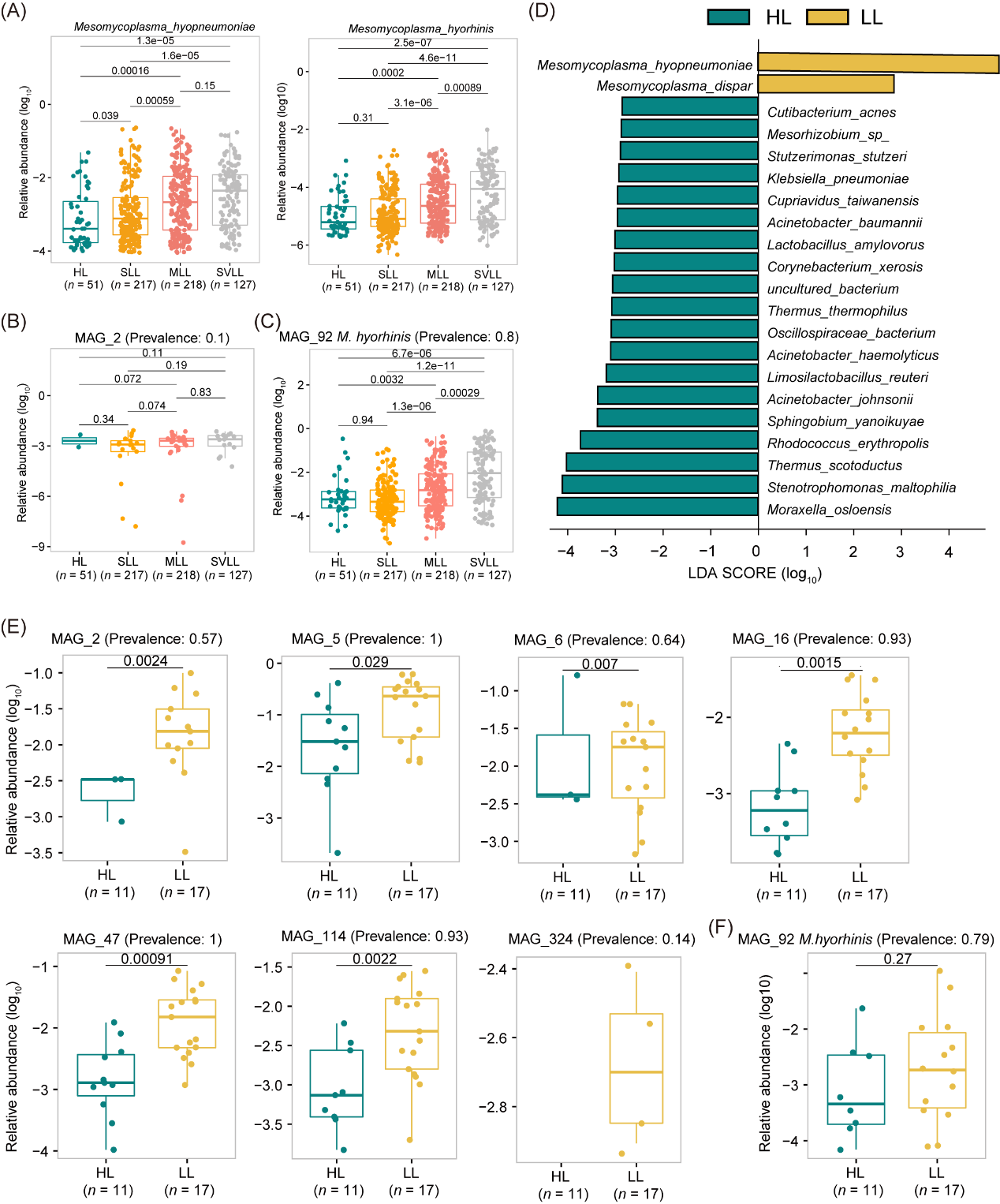


**Figure S7 Association between the lung microbiome and lung lesions.** (A) Comparison of the relative abundances of two *Mesomycoplasma spp.* among four pig groups with different severity of lung lesions. Healthy lung (HL, dark green), slight lung lesions (SLL, orange), moderate lung lesions (MLL, light red), severe lung lesions (SVLL, light grey). (B, C) Associations of *Mesomycoplasma hyopneumoniae* MAG_2 and *Mesomycoplasma* *hyorhinis* MAG_92 with lung lesions. Wilcoxon tests were used for the comparisons. The *p* values are given on the horizontal lines. The prevalence of MAGs in tested samples is shown in the bracket above boxplot. The sample number for each group is listed under the *X*-axis. (D) Validating the association of *M. hyopneumoniae* with lung lesions in the Berkshire × Licha cross line population. Yellow bars indicate the species enriched in lung-lesion pigs (LL), and dark green bars show the species enriched in healthy pigs (HL). (E) Validating the associations of *M. hyopneumoniae* MAGs with lung lesions in the Berkshire × Licha cross line population. The detailed descriptions were as same as (B, C).


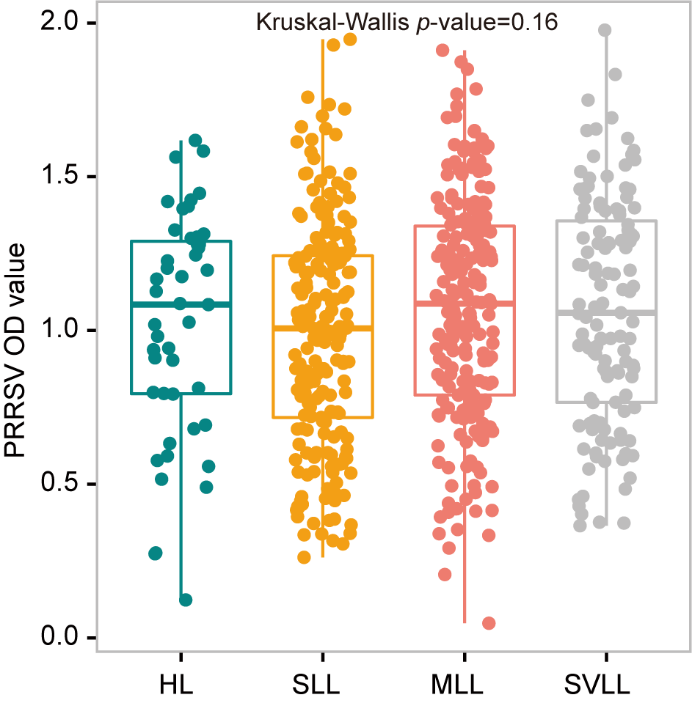


**Figure S8** **The comparison of the porcine reproductive and respiratory syndrome virus (PRRSV) antibody levels in serum samples among healthy lung, slight lung lesion, moderate lung lesion, and severe lung lesion pigs.** PRRSV antibody levels were measured using ELISA kit. The comparison was performed by the Kruskal-Wallis rank sum test. The *p*-value were given in the box-plot.


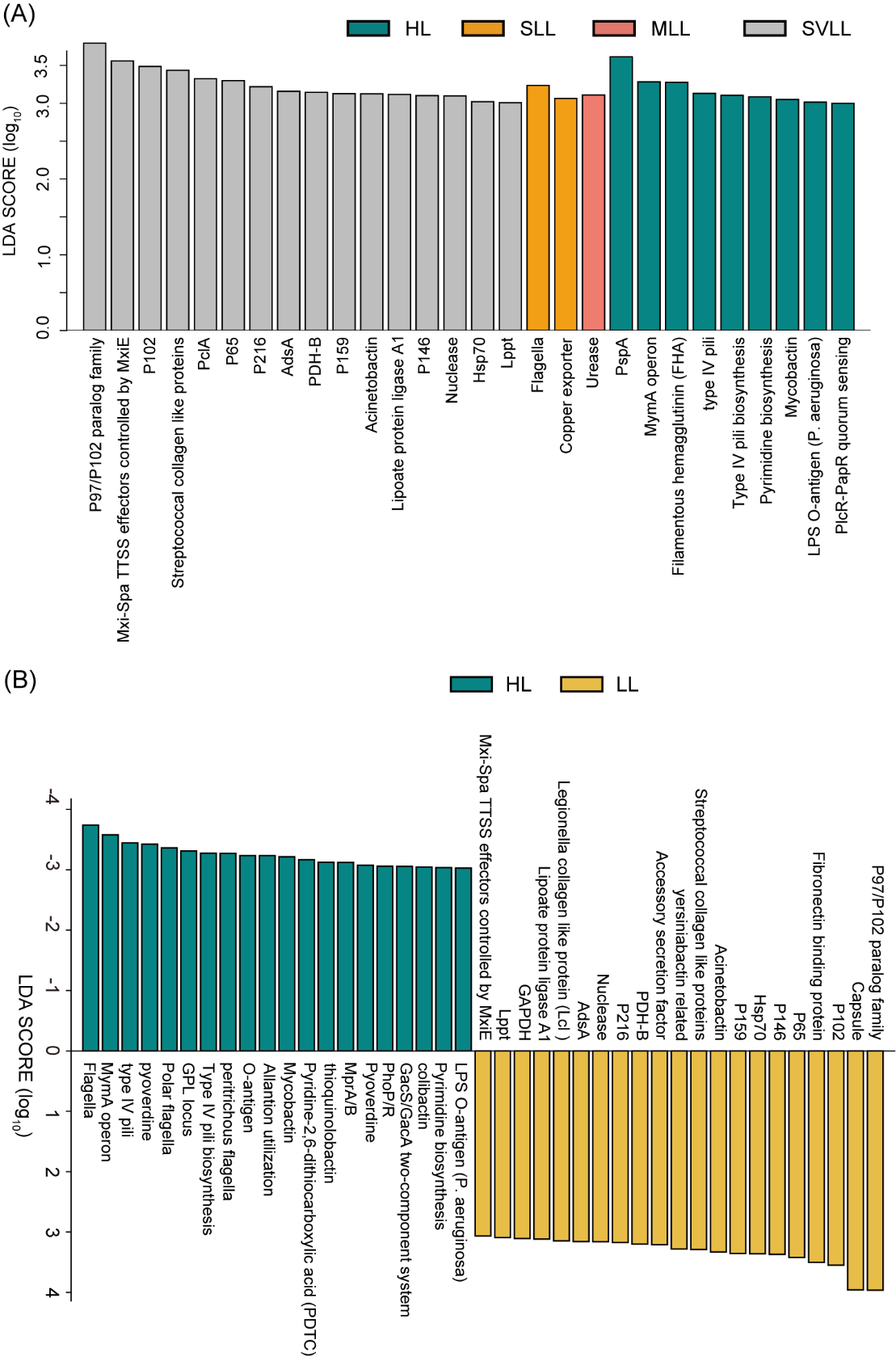


**Figure S9** **Associations of potential functional capacities of the lung microbiome with lung lesions in the F_7_ population and** **the Berkshire × Licha cross lines.** (A) Linear discriminant analysis effect size (LEfSe) analysis identified virulence factor types enriched in each group of F_7_ pigs. Healthy lung (HL, dark green), slight lung lesions (SLL, orange), moderate lung lesions (MLL, light red), severe lung lesions (SVLL, light grey). (B) identifying virulence factor types showing differential enrichments in the lung microbiome between healthy (HL) and lung lesions pigs (LL) in the Berkshire × Licha cross lines. Yellow bars indicate the items enriched in lung-lesion pigs, and dark green bars show the items enriched in healthy pigs.


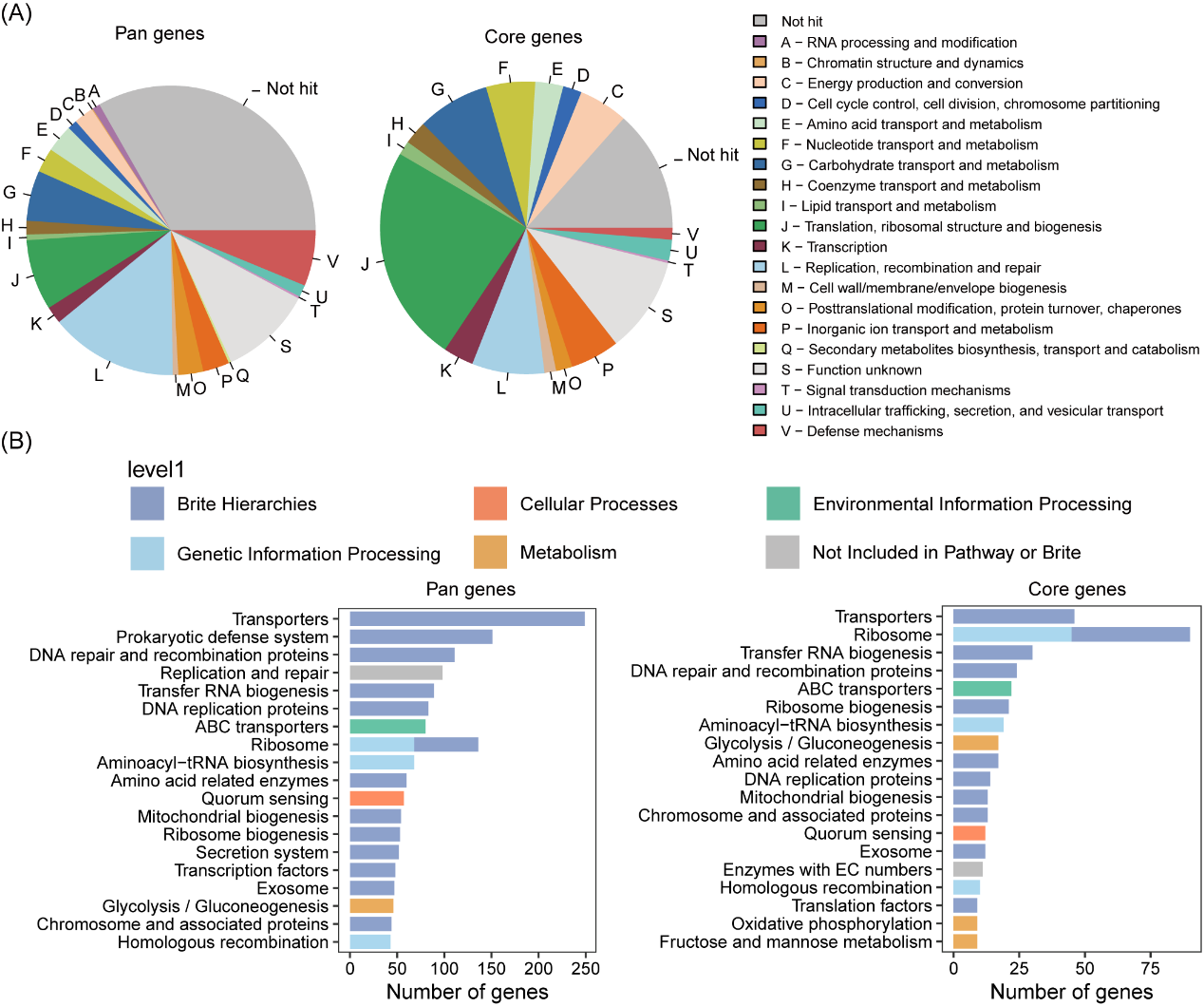


**Figure S10 Functional annotations of** ***M. hyopneumoniae* pan-genomes.** (A) The clusters of orthologous groups (COG) of pan-genes and core genes. Not hit represents the genes could not be aligned to any COG category in the database. (B) KEGG annotations for pan-genes and core genes. The *x*-axis indicates the number of genes annotated to each KEGG pathway. The bar colors indicate the categories of the KEGG pathways at the level one.


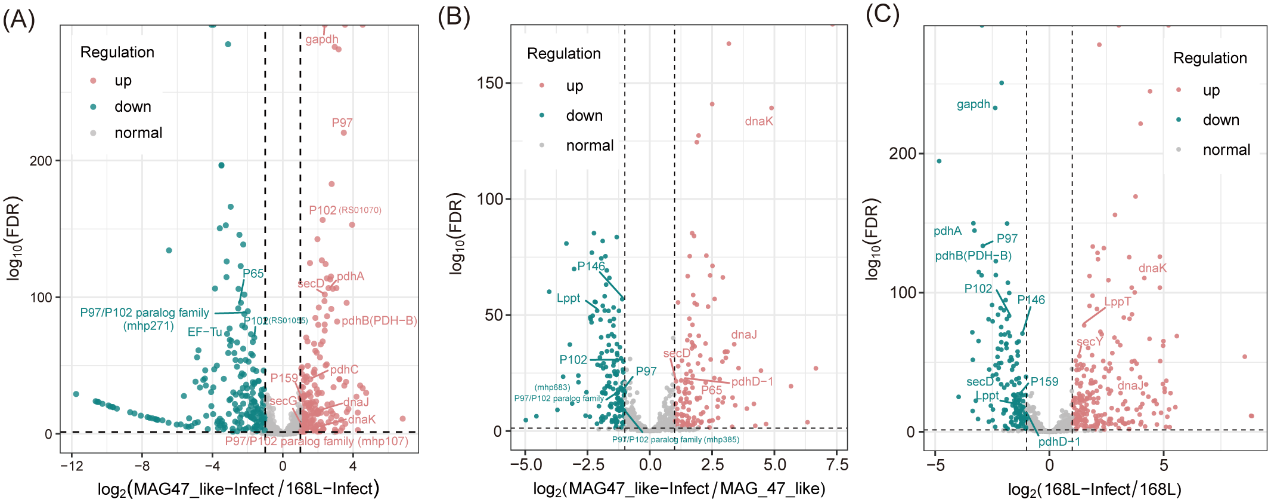


**Figure S11 Identification of differentially expressed genes (DEGs) between MAG47_like and 168L strains at 24hpi, and between bacterial cells infecting host bronchial epithelial cells (BECs) and pure cultured bacterial cells.** (A) Volcano plot showing DEGs between MAG47_like and 168L strains at 24hpi. Green dots and red dots indicate downregulated and upregulated DEGs in the MAG47_like stain compared to the 168L strain. Gray dots represent unchanged genes. (B) DEGs between bacterial cells infecting host BECs and pure cultured bacterial cells with MAG47_like strain. (C) DEGs between bacterial cells infecting host BECs and pure cultured bacterial cells with 168L strain. FDR-corrected two-sided *p*-values were calculated with the DeSeq2 in R package.


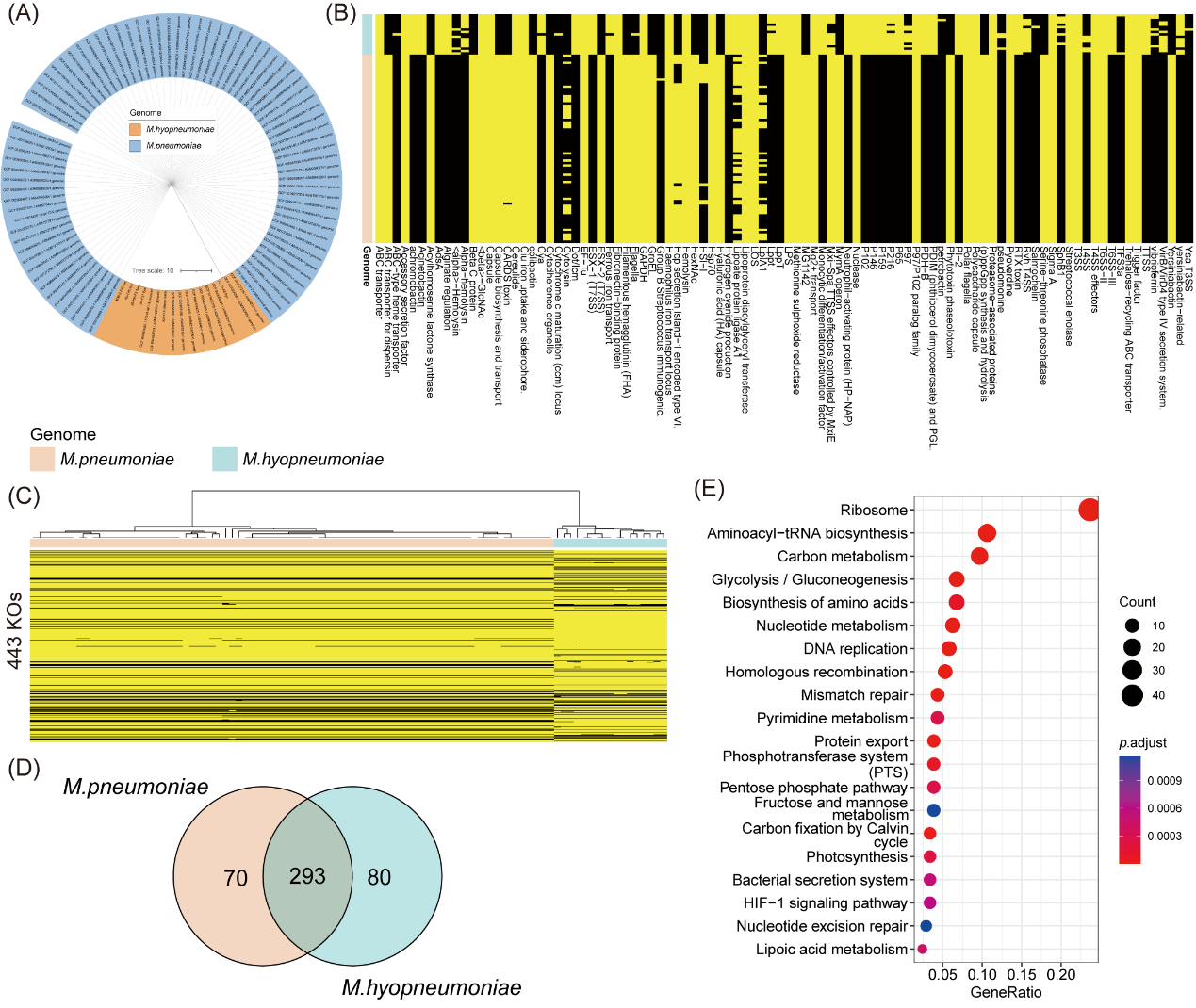


**Figure S12 Comparison of the genomic structures between *M. pneumoniae* and *M. hyopneumoniae*.** (A) Phylogenetic tree of *M. pneumoniae* and *M. hyopneumoniae* genomes. (B) and (C) The distribution of virulence factor (VF) types and KEGG ortholog (KOs) in *M. pneumoniae* (orange pink bar) and *M. hyopneumoniae* (light blue) genomes. (D) Numbers of shared or species-specific KOs between *M. pneumoniae* and *M. hyopneumoniae*. (E) KEGG pathways enriched by shared KOs.


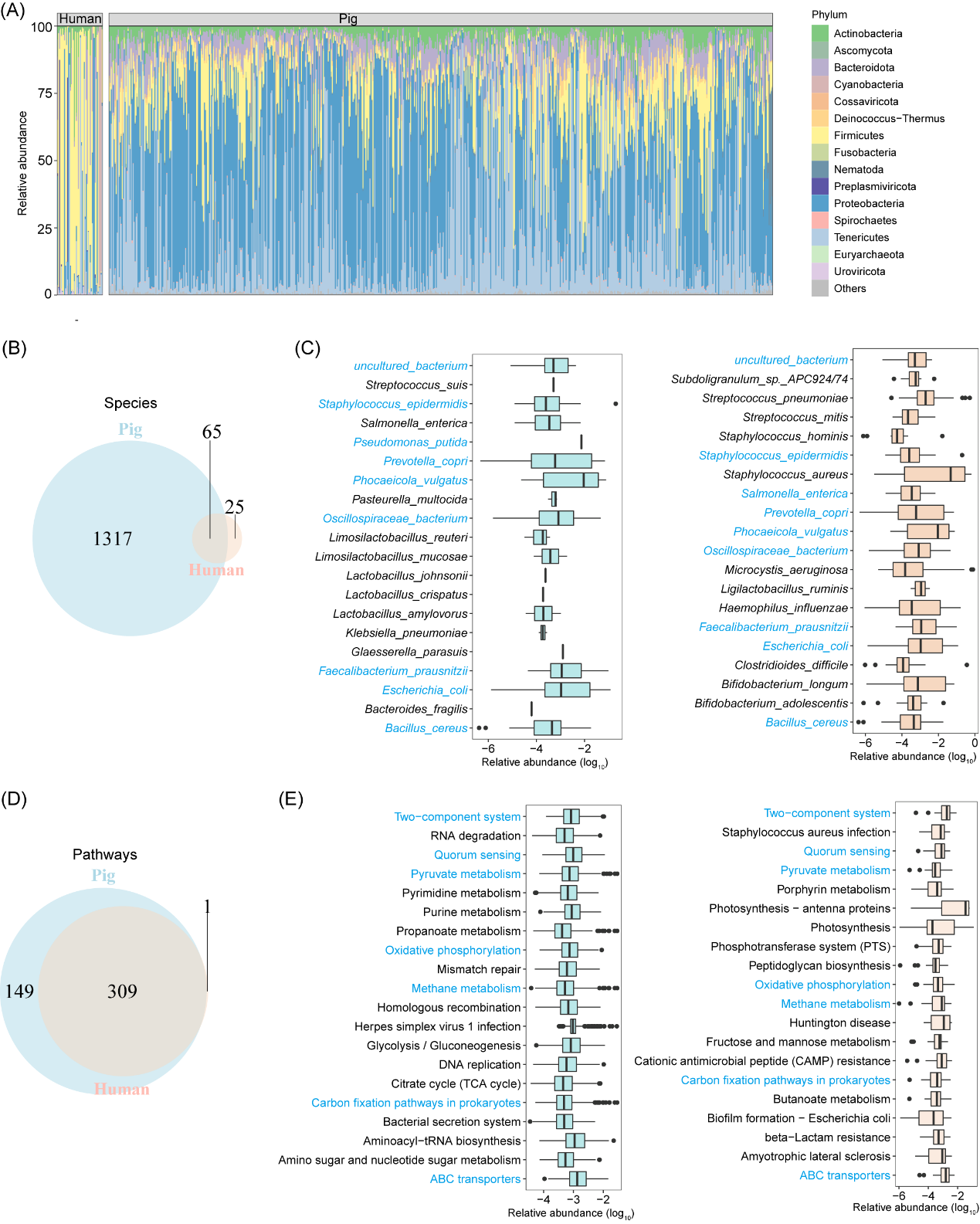


**Figure S13** **Comparison of microbial compositions and potential functional capacities of the lung microbiome between humans and pigs.** (A) The relative abundance of each bacterial phylum in the lung microbial samples of 46 children with *Mycoplasma pneumoniae* pneumonia and 670 pigs with lung lesions. (B) Pie chart indicating the numbers of core species (existing > 95% samples) shared or specifically enriched in the lung microbiome of humans and pigs. (C) The relative abundance of top 20 core species shared in the lung microbiome between humans and pigs. (D) Pie chart of core KEGG pathways in the lung microbiome between humans and pigs. (E) The relative abundance of core KEGG pathways with the relative abundance in the top 20 in the human lung microbiome compared to that in pigs. The KEGG pathways that exited in at least 95% samples in human or pig lung microbiome were defined as core KEGG pathways.
